# Towards expansion of the MATTS data bank with heavier elements: the influence of the wavefunction basis set on the multipole model derived from the wavefunction

**DOI:** 10.1107/S1600576724009841

**Published:** 2024-11-17

**Authors:** Vladislav Ignat’ev, Paulina Maria Dominiak

**Affiliations:** a Biological and Chemical Research Center, Faculty of Chemistry University of Warsaw Warsaw Poland; Shiv Nadar Institution of Eminence, India

**Keywords:** quantum crystallography, charge density, multipolar refinement, multipolar atom types from theory and statistical clustering, MATTS, transferable aspherical atom model, TAAM

## Abstract

The MATTS (multipolar atom types from theory and statistical clustering) data bank is an advanced tool for crystal structure refinement and properties analysis. It applies a multipole model (MM), which describes the asphericity of the atomic electron density and helps to interpret X-ray or electron diffraction data better than approaches based on the spherical atoms approximation. The generation of MATTS data involves density functional theory calculations, and until recently we used the B3LYP/6-31G** level of theory for this stage. However, it was not so clear how the wavefunction level of theory, especially the basis set used, influenced the resulting MM. This study investigates the influence of the wavefunction basis set on the resulting MM from a charge density point of view. For this purpose, we used charge density related properties, such as correlation of electrostatic potentials, atomic electron populations and average electrostatic potential values. The complex analysis reveals that, within the framework of MATTS data generation, the size of the basis set used has the most significant impact on the MM’s charge density quality, and switching from double- to triple-zeta basis sets helps notably improve the charge density related properties. This research sets the foundation for the creation of a new version of the MATTS data bank, which will be expanded to include atom types for elements heavier than Kr and selected metal complexes important for biological systems.

## Abstract 2

This study examines the quality of charge density obtained by fitting the multipole model to wavefunctions in different basis sets. The complex analysis reveals that changing the basis set quality from double- to triple-zeta can notably improve the charge density related properties of a multipole model.

## 1. Introduction

The multipolar atom types from theory and statistical clustering (MATTS) data bank (Rybicka *et al.*, 2022 ▸; Jha *et al.*, 2022 ▸) is based on the transferable aspherical atom model (TAAM), which states that an organic molecule’s atoms in the same chemical environment (in what follows, we will use the term ‘atom type’) are characterized by almost identical electron densities (Brock *et al.*, 1991 ▸; Pichon-Pesme *et al.*, 1995 ▸; Bąk *et al.*, 2011 ▸; Jarzembska *et al.*, 2012 ▸; Malińska *et al.*, 2014 ▸). MATTS, like its predecessor the University at Buffalo Pseudo­atom Databank (Dominiak *et al.*, 2007 ▸), stores averaged multipole model (MM) parameters for atom types which, later on, can be used in crystal structure refinements replacing the independent atom model (IAM) (Jha *et al.*, 2020 ▸; Jha *et al.*, 2023 ▸), in the reconstruction of the electron density and electrostatic potential of molecules (Kulik *et al.*, 2022 ▸), and in the estimation of the energy of electrostatic interactions (Kumar *et al.*, 2019 ▸). In particular, very recent fields exploring MATTS data bank applications concern 3D electron diffraction (3D ED, microED) (Gruza *et al.*, 2020 ▸; Kulik *et al.*, 2022 ▸; Olech *et al.*, 2024 ▸) and single-particle cryogenic electron microscopy (cryo-EM) (Bick *et al.*, 2024 ▸). We note that, besides MATTS, there are other multipolar parameter data banks available – the most popular are ELMAM2 (Domagała *et al.*, 2012 ▸) and Invariom (Dittrich *et al.*, 2013 ▸).

In order to generate MATTS data, *i.e.* to obtain multipole parameters for certain atom types, we fit the MM parameters to the simulated X-ray diffraction pattern of a ‘model’ mol­ecule containing such an atom type, inserted in a pseudo-cubic unit cell. The fitting process is basically a crystallographic refinement which applies the Hansen–Coppens pseudo-atom model (Hansen & Coppens, 1978 ▸) [equation (1)] to describe the aspherical electron density of the atoms (and hence the scattering),
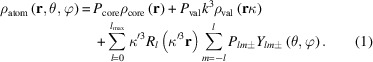
Here, the first term is the spherical core electron density, the second term is the spherical valence electron density and the third term is the aspherical valence electron density of the atom.

One of the most important parameters which can be varied during MM construction is the data bank of radial functions used. Data banks of radial functions use a pre-derived analytical expression to compute the electron density of atoms [ρ_core_(**r**) and ρ_val_(**r**)] and the scattering factors. The last published version of MATTS (Rybicka *et al.*, 2022 ▸; Jha *et al.*, 2022 ▸) uses a data bank based on Clementi–Roetti non-relativistic wavefunctions (Clementi & Roetti, 1974 ▸). However, the Clementi–Roetti data bank is available only for elements up to Kr. This means that application of the Clementi–Roetti data bank prevents us from expanding MATTS to heavier elements widely represented in organic crystals and biomolecules, such as iodine. That is why, in this work, we test a radial function data bank based on Su–Coppens–Macchi relativistic wavefunctions, which are available for elements up to Xe (Su & Coppens, 1998 ▸; Macchi & Coppens, 2001 ▸).

The aforementioned diffraction pattern is simulated by single-point density functional theory (DFT) calculations which are, in turn, based on the experimental geometries of a model molecule (Dominiak *et al.*, 2007 ▸) taken from the Cambridge Structural Database (Groom *et al.*, 2016 ▸) or Protein Data Bank (Berman *et al.*, 2000 ▸). Until recently we used the B3LYP/6-31G** combination for the single-point calculation step. This combination showed good compatibility with the MM, which has been proven by both the accuracy of the electron densities (Volkov *et al.*, 2007 ▸) and related properties, such as electrostatic interaction energies (Volkov *et al.*, 2006 ▸; Kumar *et al.*, 2019 ▸). However, when applying the 6-31G** basis set in the MATTS data bank we face the same limitations as with Clementi–Roetti radial functions. Elements heavier than Kr are unavailable in 6-31G**, as well as in bigger Pople basis sets (Francl *et al.*, 1982 ▸; Krishnan *et al.*, 1980 ▸). Moreover, bigger basis sets from the Pople family are inferior in the quality of their valence functions compared with other popular basis sets (Grev & Schaefer, 1989 ▸).

This necessity to change the basis set of quantum-chemical calculations for MATTS data generation therefore raised a question: which combination of functional/basis set is most suitable for an MM? First, we have to note that the MATTS data set consists of a thousand molecules, and we cannot step outside the DFT framework, because methods with a higher level of theory (like post-Hartree–Fock methods) will result in unacceptable computational cost. The old hybrid generalized gradient approximation functionals, such as B3LYP, are still the most accurate among other DFT functionals in both electron density distribution and energy calculations, despite the fact that, since their inception, many new DFT functionals have been created. These modern highly parameterized functionals often underperform B3LYP in terms of accuracy of electron density and derived properties distribution (Medvedev *et al.*, 2017 ▸; Hait & Head-Gordon, 2018 ▸). That is why we abstained from thorough testing of functionals.

Next, when we narrowed down our study of basis set investigations, due to the abundance of basis sets available we had to define a parameter for basis set comparison. For this parameter we chose the number of basis functions corresponding to each valence atomic orbital (zeta-quality of basis set) and then we focused on the most popular Gaussian-type basis set families (Table 1 ▸). Together with the previously used Pople 6-31G** (Francl *et al.*, 1982 ▸) we examined double- and triple-zeta basis sets from the Ahlrichs family (Def2SVP and Def2TZVP) (Schäfer *et al.*, 1994 ▸; Gulde *et al.*, 2012 ▸) and double- and triple-zeta basis sets from the Dunning family, also known as the correlation consistent family (in fact we used a combination of pseudopotential basis sets for heavy atoms and standard basis sets for all other atoms: cc-pVDZ-PP/cc-pVDZ and cc-pVTZ-PP/cc-pVTZ) (Dunning, 1989 ▸; Prascher *et al.*, 2010 ▸; Peterson *et al.*, 2003 ▸). As the reference point, we used calculations based on the universal all-electron UGBS1P basis set introduced by de Castro & Jorge (1998 ▸).

In order to examine the quality of MM fitting to wavefunctions based on different basis sets, we compared the resulting MM and wavefunction based on the UGBS1P basis set (the reference) using charge density related parameters. For this purpose, we used the correlation between electrostatic potentials, atomic electron populations, electrostatic potential values averaged over molecular surfaces and crystallographic *R* factors [equation (2)] as parameters for comparison.
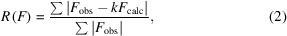
where *F*_obs_ are structure factors computed from the molecular wavefunction, *F*_calc_ are structure factors computed from the MM and *k* is a scale factor.

To estimate the correlation of electrostatic potentials, we used Pearson correlation coefficients between electrostatic potential grids, 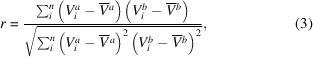
where *V*^*a*^ is the point electrostatic potential of grid *A*, *V*^*b*^ is the point electrostatic potential of grid *B* and 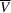 is the average electrostatic potential.

For atomic electron populations we used the quantum theory of atoms in molecules (QTAIM) (Bader & Nguyen-Dang, 1981 ▸; Bader, 2005 ▸) definition. According to QTAIM the space around a molecule can be partitioned into atomic basins, each basin being defined by its own set of trajectories traced out by the gradient vectors of the density (∇**r**) that terminate at a given nucleus. The number of electrons *N*_a_ in an atom can be computed by integrating the electron density ρ(**r**) over its atomic basin,
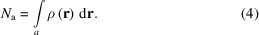
In order to investigate the electrostatic potential averaged over the molecular surface, we used an approach developed by Politzer & Murray (2002 ▸). The electrostatic potential *V*(**r**) that the nuclei and electrons of a molecule create at any point **r** in the surrounding space can be described as
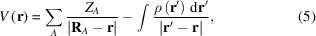
where *Z_A_* is the nucleus charge, **R***_A_* is the nucleus position and ρ(*r*) is the electron density. The sign of *V*(**r**) shows which term of the equation (‘nucleus’ or ‘electron density’ term) contributes more at the point **r**. Averaging *V*(**r**) values over a certain molecular surface (iso-contours of electron density plotted at levels of 0.1 a.u., 0.01 a.u. *etc.*) we can obtain an averaged electrostatic potential of the surface,
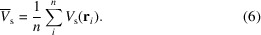


At the end of this introduction, we note that the topic of MM fitting to theoretical electron densities has been addressed in plenty of studies. Koritsanszky *et al.* (2012 ▸) discussed MM fitting to theoretical structure factors based on DFT and post-Hartree–Fock wavefunctions of isolated mol­ecules, noting that a wavefunction’s level of theory influences the fitting results. Bąk *et al.* (2012 ▸) investigated how well a fitted MM can reproduce certain electron density and electrostatic properties of the original wavefunction of an isolated molecule. However, there was no systematic study of the impact of a wavefunction’s basis set on the electrostatic properties of a fitted MM.

## 2. Methods

### 2.1. Charge density in terms of MM

**Figure 1:**
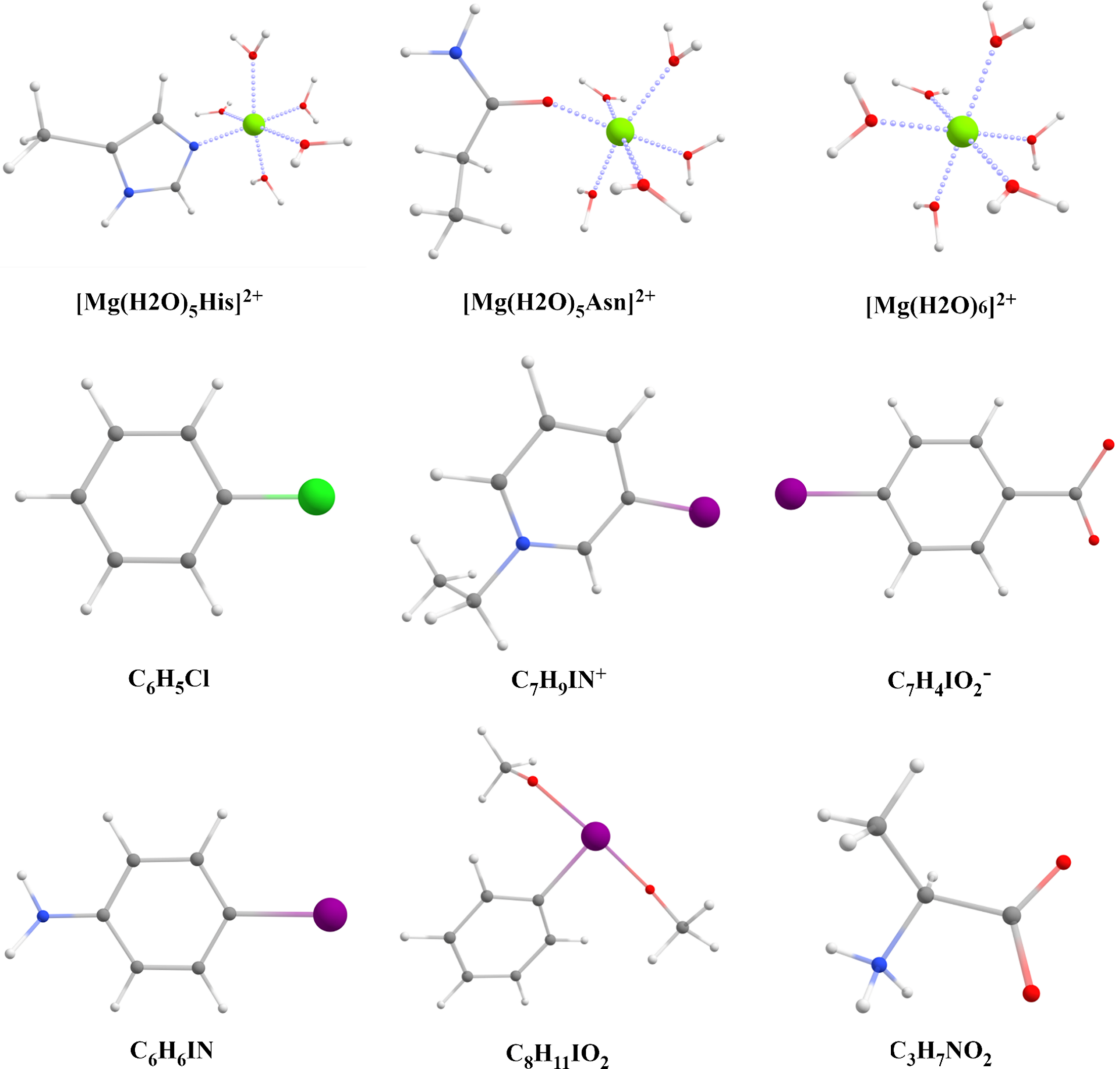
The molecules under study. PDB IDs and residue number, or CSD refcodes, are as follows: [Mg(H_2_O)_5_His]^2+^ (1f97, H_1f97_1_250_A; Kostrewa *et al.*, 2001 ▸), [Mg(H_2_O)_5_Asn]^2+^ (1e42, H_1e42_3_960_A; Owen *et al*., 2000 ▸), [Mg(H_2_O)_6_]^2+^ (2wfi, H_2wfi_1_1002_A; Stegmann *et al.*, 2009 ▸), C_6_H_5_Cl (MCBENZ03; Nath & Naumov, 2015 ▸), C_7_H_9_IN^+^ (CAGNOR; Fotović & Stilinović, 2021 ▸), C_7_H_4_IO_2_^−^ (CAJCAV; Archana *et al.*, 2021 ▸), C_6_H_6_IN (EJAYET; Dey *et al.*, 2003 ▸), C_8_H_11_IO_2_ (JAMJIU; Ghosh *et al.*, 2021 ▸) and C_3_H_7_NO_2_ (LALNIN03; Destro *et al.*, 1991 ▸).

To model the charge density in terms of MM we chose nine model structures (Fig. 1 ▸) from the Cambridge Structural Database (CSD) (Groom *et al.*, 2016 ▸) or Protein Data Bank (PDB) (Berman *et al.*, 2000 ▸) and used their experimental geometries for DFT calculations. In the case of structures taken from the PDB {[Mg(H_2_O)_5_His]^2+^, [Mg(H_2_O)_5_Asn]^2+^ and [Mg(H_2_O)_6_]^2+^} we used only a fragment of the whole protein molecule and optimized the hydrogen positions in the *GAUSSIAN16* program (Frisch *et al.*, 2016 ▸) using the B3LYP/6-31G** level of theory. In the case of structures taken from the CSD (C_6_H_5_Cl, C_7_H_9_IN^+^, C_7_H_4_IO_2_^−^, C_6_H_6_IN, C_8_H_11_IO_2_ and C_3_H_7_NO_2_), hydrogen positions were corrected to achieve the average *X*—H bond lengths observed in neutron diffraction (Allen & Bruno, 2010 ▸) following the procedure used to build the MATTS data bank.

The wavefunctions of the model structures were obtained by single-point calculations in the B3LYP functional and basis set under study using *GAUSSIAN16*.

Theoretical static structure factors in the range 0 < sin(θ/λ) < 1.1 Å^−1^ were obtained by analytical Fourier transform of the model structure’s wavefunction (excluding the molecular orbitals of core electrons) for reciprocal-lattice points corresponding to a pseudo-cubic cell with 30 Å edges. The resulting valence-only structure factors were fitted with the Hansen–Coppens multipole formalism using multipolar refinement with the *XD16* program suite (Volkov *et al.*, 2016 ▸). The Clementi–Roetti (Clementi & Roetti, 1974 ▸) and Su–Coppens–Macchi (Su & Coppens, 1998 ▸; Macchi & Coppens, 2001 ▸) radial function data banks were used during the refinement. Refinement was carried out only on the MM parameters [equation (1); *i.e.* atomic coordinates were not refined and the scale factor was kept equal to 1.0]. The multipole levels of the atoms were expanded up to hexadecapoles (except for hydrogen atoms, for which the multipole expansion was truncated up to quadrupoles; discussion of this issue is provided in Section S1 of the supporting information). No symmetry restrictions were put on atomic MM parameters during refinement {with a few exceptions: in all molecules, only bond-oriented multipoles were refined for hydrogen atoms, and the symmetry-equivalent atoms of the [Mg(H_2_O)_6_]^2+^ and C_6_H_5_Cl molecules shared the same set of multipole populations to stabilize the refinement}. No restrictions were put on the κ and κ′ parameters. Individual κ and κ′ parameters for each atom were used (except for hydrogen atoms, where we used the joined κ and κ′ for chemically identical hydrogens). No atomic displacement parameters were included in the structure factor calculation step nor during the refinement. Also, we emphasize that, since we omitted core electrons at the structure factor calculation stage, the refined MM does not contain core electrons either.

**Figure 2:**
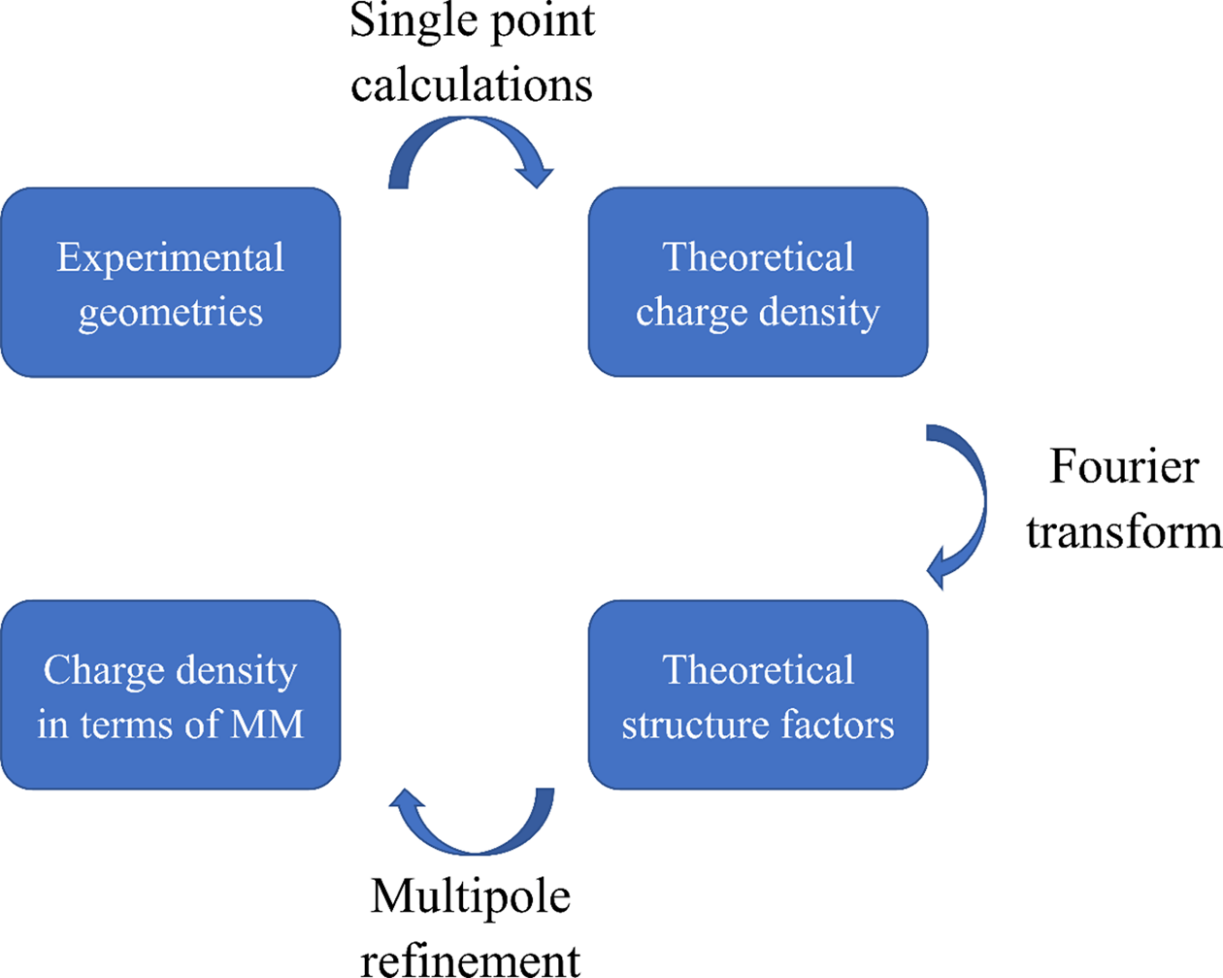
An approach to retrieving charge density in terms of MM for experimentally determined structures.

A simplified scheme of this method is shown in Fig. 2 ▸.

### 2.2. Grids of electron density and electrostatic potential

The charge density properties mentioned below were calculated using grids of electron density and electrostatic potential. Since we carried out an analysis of comparably sized molecules we used unified options for grids of different molecules, and unified options were used for electron density and electrostatic potential grids as well. We used cubic grids with dimensions of 25.416734 bohr [step of 0.094486 bohr (approximately 0.05 Å), 270 steps in each direction, total number of grid points 19 683 000]. Grids were generated in the *Multiwfn* program (Lu & Chen, 2012 ▸) for wavefunctions and in *XD16* for multipole and independent atom (superposition of spherical atomic electron densities) models. In the IAM case, multipole populations (*l* ≥ 1) were set to zero, monopole populations were set to the free atom values and the κ parameters were set to unity. For grid calculations, the valence-only MM and IAM were expanded back with core electrons. Extra care has been taken to obtain grids from these two programs that are oriented in exactly the same way with respect to the atom positions of a given molecule.

### 2.3. Correlation coefficients

In order to investigate the correlation between the electro­static potential of the system under study (wave­function, or MM based on a particular wavefunction) and the electrostatic potential of the reference (wavefunction in the UGBS1P basis set) we calculated Pearson correlation coefficients [equation (3)] between grids of electrostatic potential.

### 2.4. QTAIM atomic electron populations

Topological analysis of the electron density of the system under study (wavefunction, or MM based on a particular wavefunction) and the electron density of the reference (wavefunction in the UGBS1P basis set) was carried out on grids of electron density in *Multiwfn*. Topological analysis was performed as follows: the location of critical points and generation of basins were carried out on the reference grid, then integration over the generated basins was carried out on both the reference grid and the grid of the system under study (*i.e.* we integrated the electron density over unified basins). This procedure allowed us to perform a quantitative analysis of the charge distribution in 3D space (reduced to atomic electron populations) that was not affected by differences in the definition of atomic basin boundaries. The difference between the electron populations of identical atoms in the system under study (*N_i_*) and in the reference (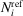) is described as
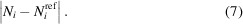
Note that the difference between the atomic electron populations is equal to the difference in atomic net charges 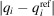.

### 2.5. Molecular surface electrostatic potentials

To analyse electrostatic potentials on molecular surfaces we used the approach developed by Politzer & Murray (2002 ▸). For each of the test molecules we built a set of molecular electron density surfaces (namely 0.1, 0.05, 0.01, 0.001 and 0.0001 a.u. surfaces) in the reference (wavefunction in the UGBS1P basis set) electron density grid and mapped the electrostatic potential grid from the system of interest (wavefunction, or MM based on a particular wavefunction) onto them. Then we averaged the electrostatic potential values over the molecular surfaces. This analysis of molecular surfaces was carried out in *Multiwfn*.

In order to evaluate the average electrostatic potential difference between the surfaces of the system under study and the reference we used averaged error metrics: mean error ME [equation (8)], mean absolute error MAE [equation (9)] and root-mean-square error RMSE [equation (10)]:
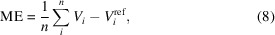

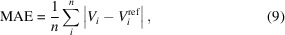

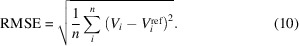
We use several metrics because each of them has its own advantages and disadvantages. The ME is the simplest and may show the direction of systematic error. The MAE is as easy to interpret as the mean error but is not affected by the sign of residuals, so it is less prone to error cancellation than ME. Finally, the RMSE is the least straightforward one but the most sensitive to outliers and is often considered as the most statistically valid.

Usually [but not always; Roos & Murray (2024 ▸)], in work dedicated to the analysis of molecular surface properties (Brinck *et al.*, 2003 ▸; Bader *et al.*, 1987 ▸), the authors consider low-value surfaces close to the van der Waals radii of the molecule (0.001 a.u., 0.002 a.u. *etc.*), since analysis of such iso-density surfaces helps in the investigation of intermolecular interactions. However, since we are interested in the quality of the whole charge density and electrostatic potential ‘recreated’ by the MM, we extended the range of iso-density surfaces being studied.

## 3. Results and discussion

### 3.1. Consequences of changing radial functions from Clementi–Roetti to Su–Coppens–Macchi

**Figure 3:**
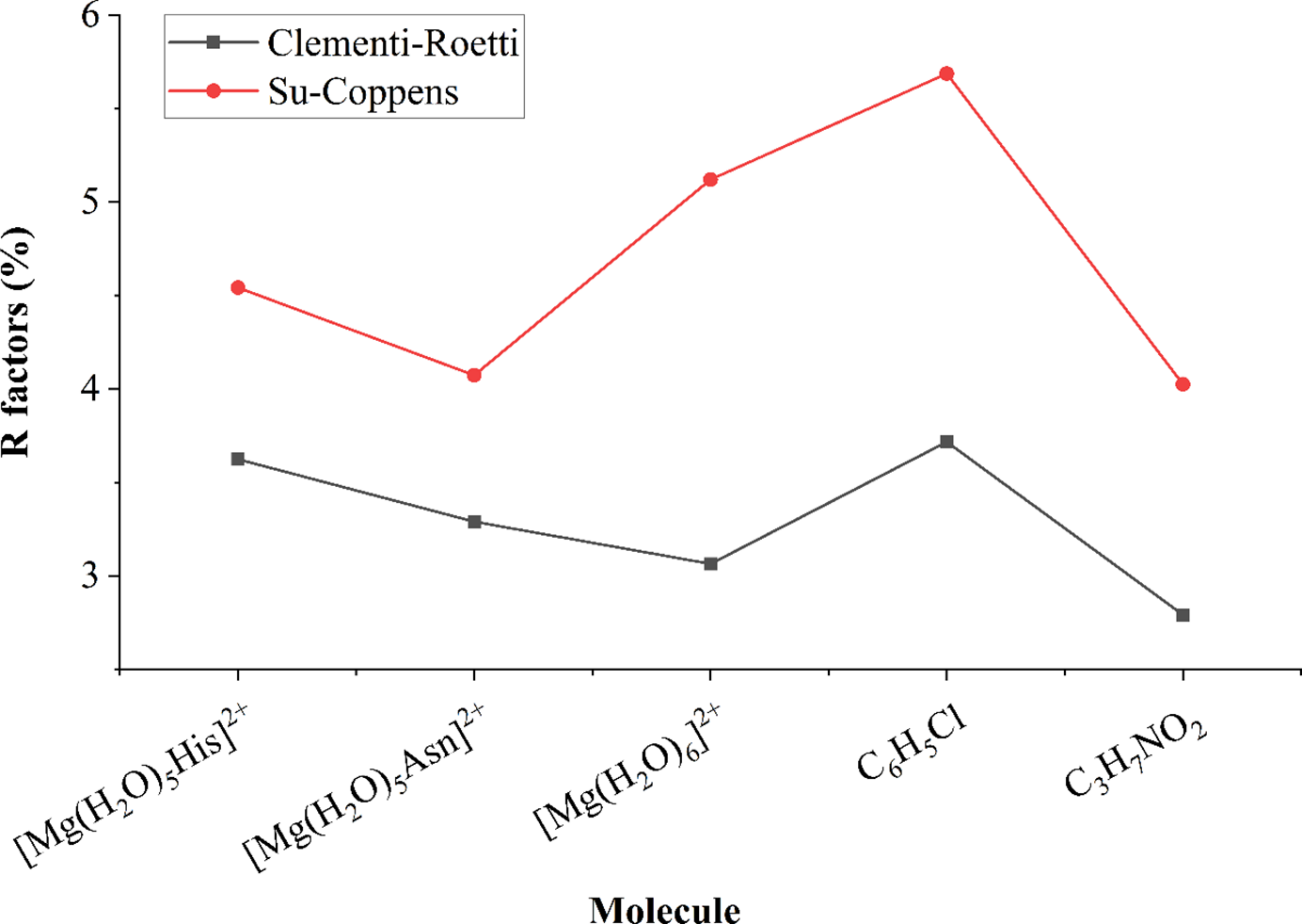
*R* factors of MMs refined against the structure factors based on wavefunctions in the 6-31G** basis set (molecules containing iodine are excluded due to limitations of the 6-31G** basis set, see Table 1).

When we compare the Clementi–Roetti (CR) and Su–Coppens–Macchi (SC) radial function data banks, usage of the SC data bank during the refinement results in worse agreement between the electron density of the resulting MM and the structure factors (higher *R* factor) in most cases. As an example, we provide a refinement of the structure factors based on wavefunctions in the 6-31G** basis set (Fig. 3 ▸).

**Figure 4:**
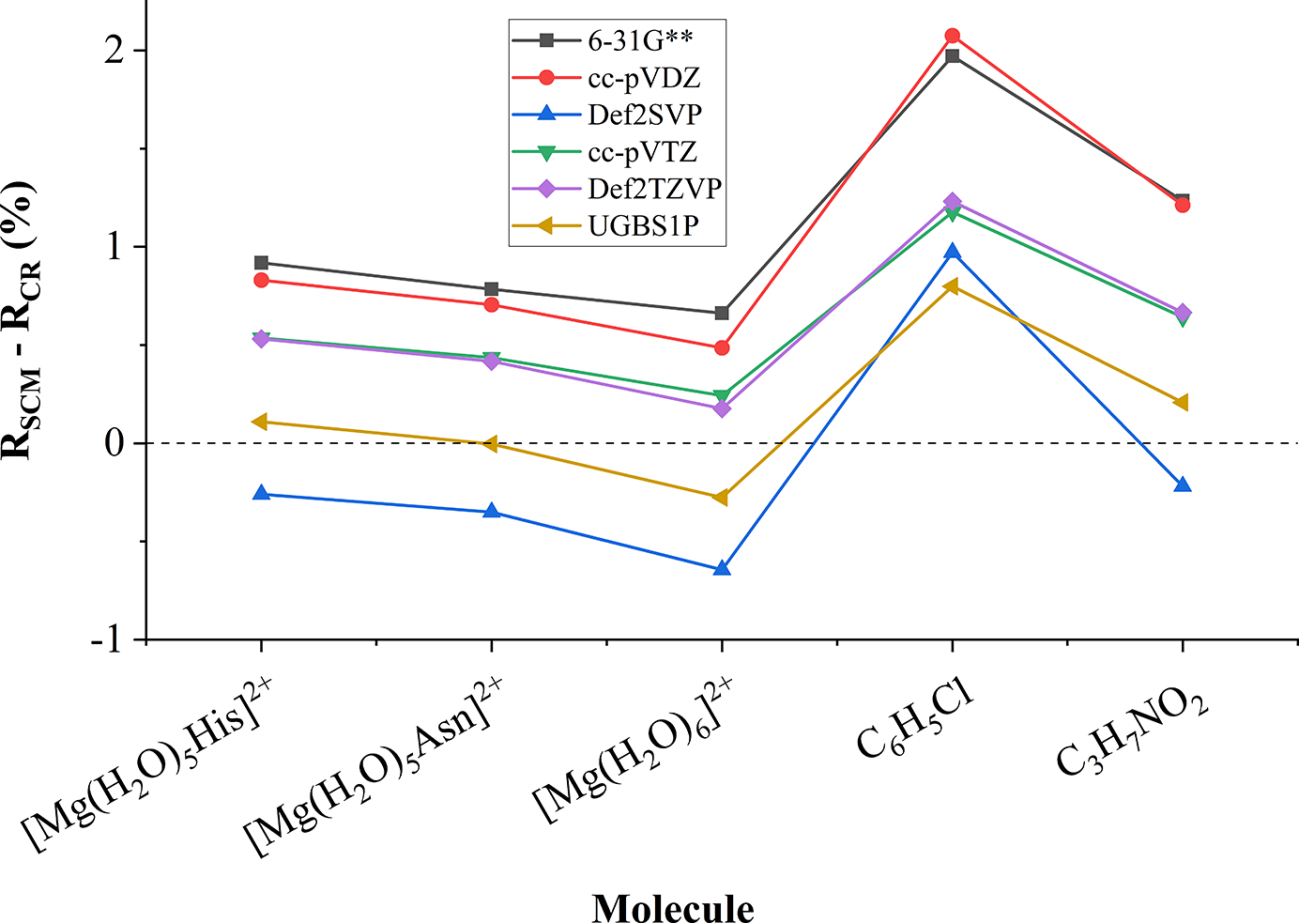
Difference between SC and CR data banks in terms of *R* factors of MMs refined against the structure factors based on wavefunctions in different basis sets (molecules containing iodine are excluded due to limitations of the 6-31G** basis set, see Table 1).

The biggest difference between the CR and SC data banks is associated with the double-zeta basis sets 6-31G** and cc-pVDZ. When we move from smaller to bigger basis sets (double-zeta basis sets 6-31G**, Def2-SVP, cc-pVDZ → triple-zeta basis sets Def2-TZVP, cc-pVTZ → UGBS1P), we can observe a gradual decrease in the difference between the CR and SC data banks in terms of *R* factors (Fig. 4 ▸). The only outlier is the Def2-SVP basis set, which often shows a difference comparable to triple-zeta basis sets. In all cases application of the biggest basis set, UGBS1P, results in the lowest difference between CR and SC *R* factors.

MMs constructed with the CR or SC data banks use the same set of radial functions and spherical harmonics for aspherical valence density [equation (1), *R*_*l*_ and *Y*_*lm*_] (Clementi & Raimondi, 1963 ▸), and since we use valence-only structure factors, we exclude the radial functions of the spherical core density [equation (1), ρ_core_] from the refinement procedure. In other words, the difference which we see in the results of application of the CR and SC data banks in our approach is caused only by the radial functions of the spherical valence density [equation (1), ρ_val_]. Nevertheless, since *P*_val_, κ, κ′ and *P*_*lm*_ are refinable parameters, the set of radial functions used in ρ_val_ influences the values of parameters for both the spherical and aspherical parts of the valence density.

We note that both CR and SC radial function data bank parameters used for spherical core and spherical valence density (coefficients and exponents of Slater-type functions) originate from analytical atomic wavefunctions. The main difference is that the SC data bank parameters are based on relativistic Dirac–Fock calculations in Slater-type orbitals (Su & Coppens, 1998 ▸; Macchi & Coppens, 2001 ▸), while the CR data bank parameters originate from non-relativistic Roothaan–Hartree–Fock calculations in Slater-type orbitals (Clementi & Roetti, 1974 ▸). We therefore suggest that the higher *R* factors associated with application of the SC data bank are caused by the fact that we are fitting advanced relativistic analytical expressions based on Slater-type functions to structure factors based on more primitive non-relativistic DFT wavefunctions in Gaussian-type functions.

Returning to the main topic, the size of the basis set used for the wavefunction calculation stage significantly influences the resulting MM, and switching from the CR to the SC data bank may require the application of basis sets with quality higher than double-zeta.

From now on, we will discuss only the results of the refinement based on the SC data bank.

### 3.2. Influence of basis set size on resulting MM charge density

**Figure 5:**
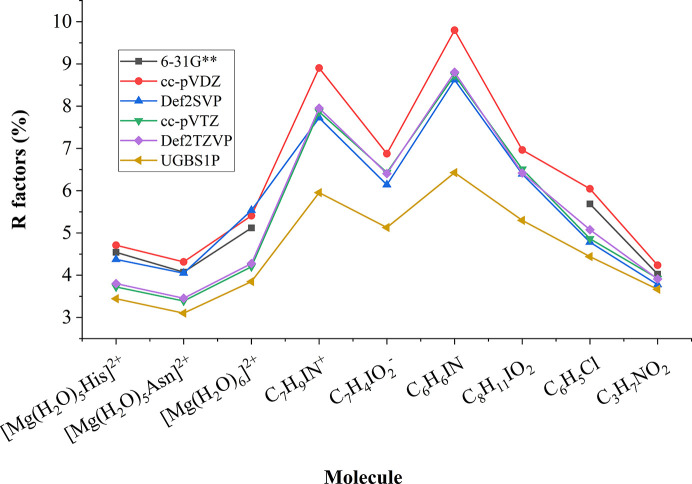
*R* factors of MMs refined against the structure factors based on wavefunctions in different basis sets (molecules containing iodine do not have data related to the 6-31G** basis set due to limitations of the basis set, see Table 1).

**Figure 6:**
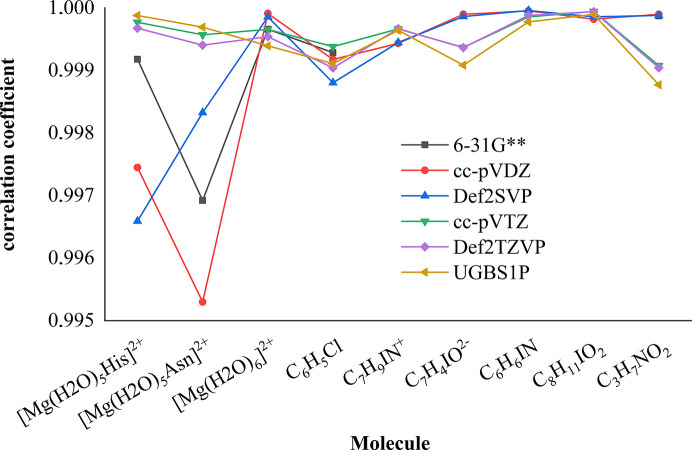
Pearson correlation coefficients between the electrostatic potential of an MM based on wavefunction in a particular basis set and the electrostatic potential of a wavefunction in the UGBS1P basis set.

If we take a look at the *R* factors obtained after refinement of an MM against structure factors based on wavefunctions in different basis sets (Fig. 5 ▸), we see a simple trend – application of a bigger basis set for the wavefunction calculation results in better agreement between the MM and theoretical structure factors (as in the previous section, the only outlier is the Def2-SVP basis set). However, as Fig. 6 ▸ shows, the correlation coefficients between the electrostatic potential of an MM and the electrostatic potential of the reference wavefunction are not strongly dependent on the basis set used for the MM. Exceptions are the [Mg(H_2_O)_5_His]^2+^ and [Mg(H_2_O)_5_Asn]^2+^ molecules. For these structures we can observe a clear gap between the correlation coefficients of MMs based on double-zeta basis sets and the correlation coefficients of MMs based on bigger basis sets (for a discussion of correlation coefficients between the electrostatic potentials of the wavefunctions themselves and the reference wavefunction, see Section S2 in the supporting information).

Now let us move on to more detailed features of the charge density. Since we used metrics of averaged errors in the discussion dedicated to atomic electron populations and averaged electrostatic potential on molecular surfaces, in order to exclude the influence of magnesium intermolecular complexes on the general sample we decided to divide the data into two samples: intermolecular complexes of magnesium {[Mg(H_2_O)_5_His]^2+^, [Mg(H_2_O)_5_Asn]^2+^ and [Mg (H_2_O)_6_]^2+^} and molecules with covalent bonds only (C_6_H_5_Cl, C_7_H_9_IN^+^, C_7_H_4_IO_2_^−^, C_6_H_6_IN, C_8_H_11_IO_2_ and C_3_H_7_NO_2_).

**Figure 7:**
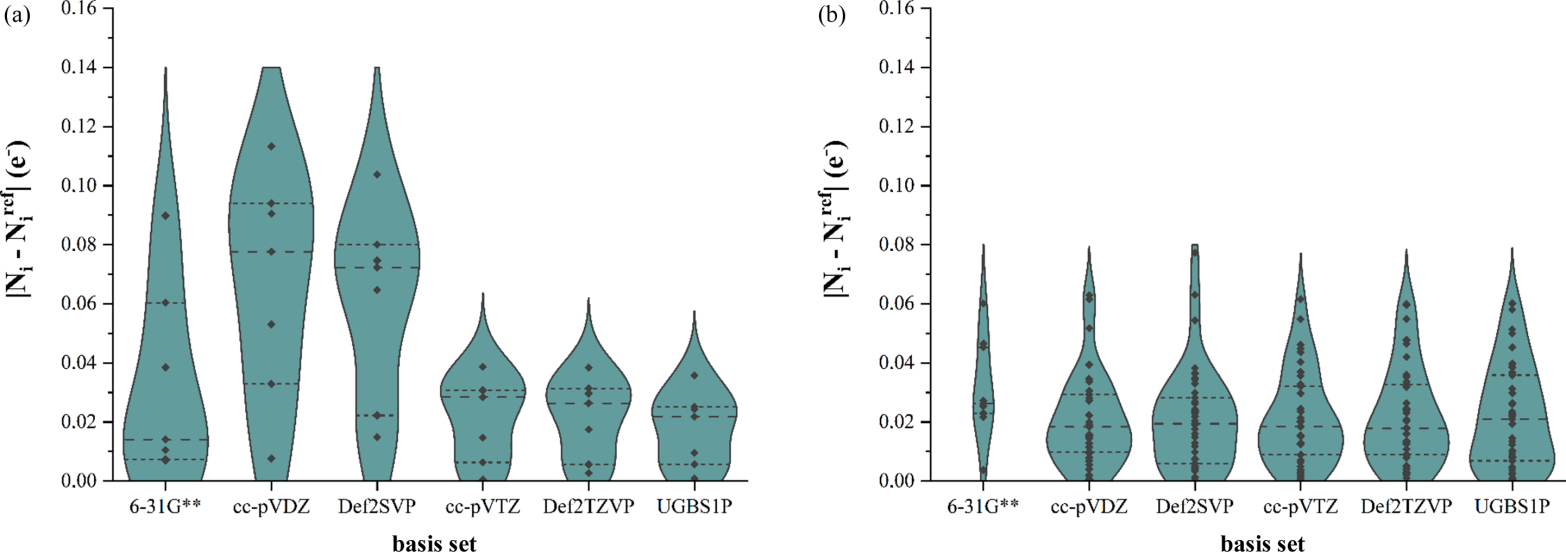
Errors in carbon atom electron populations of MMs based on different basis sets. (*a*) Intermolecular complexes of magnesium and (*b*) the rest of the molecules.

To quantify the charge distribution, we used the difference between the atomic electron populations of the MM fitted to structure factors based on wavefunctions in different basis sets (*N_i_*) and atomic electron populations of the reference wavefunction (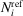). In Figs. 7 ▸ and 8 ▸ we present the differences for elements common to both samples (carbon and hydrogen, respectively). The trends are similar to what we saw in the graph related to correlation coefficients (Fig. 6 ▸). Application of double-zeta basis sets in building an MM of magnesium intermolecular complexes results in higher errors in the carbon and hydrogen electron populations than for MMs based on bigger basis sets, whereas the carbon and hydrogen electron population errors associated with structures with no intermolecular bonds are almost independent of the basis set used for the MM. We have excluded all other elements (nitrogen, oxygen, magnesium, chlorine and iodine) from this discussion, mainly due to their scarcity in the data samples. However, for completeness, analogous graphs for these other elements are given in Section S3 of the supporting information.

**Figure 9:**
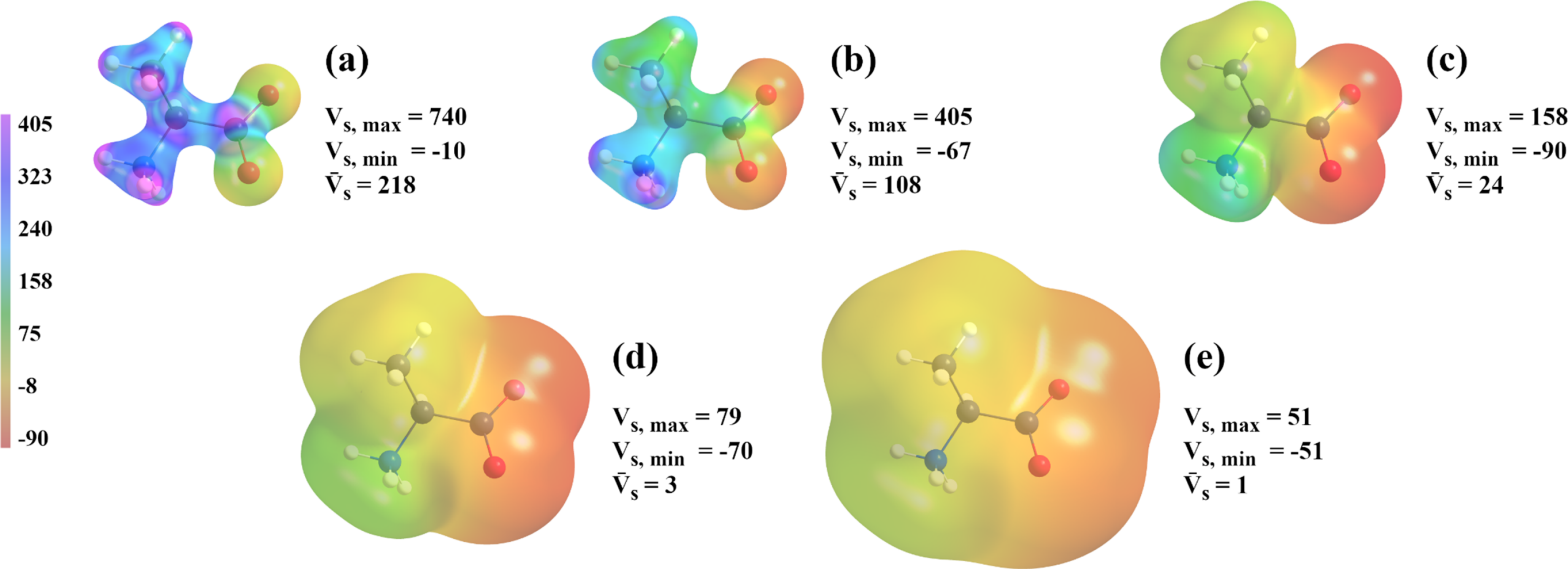
Visualization of the C_3_H_7_NO_2_ molecule iso-density surface with the reference electrostatic potential mapped onto it. Iso-density surface values are (*a*) 0.1 a.u. (surface positive electrostatic potential values truncated to 405 kcal mol^−1^ e^−1^), (*b*) 0.05 a.u., (*c*) 0.01 a.u., (*d*) 0.001 a.u. and (*e*) 0.0001 a.u.. The range of electrostatic potential values is from −90 to 405 kcal mol^−1^ e^−1^. *V*_s,max_, *V*_s,min_ and 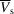 denote the maximum, minimum and average electrostatic potential values on a given surface, respectively.

**Figure 10:**
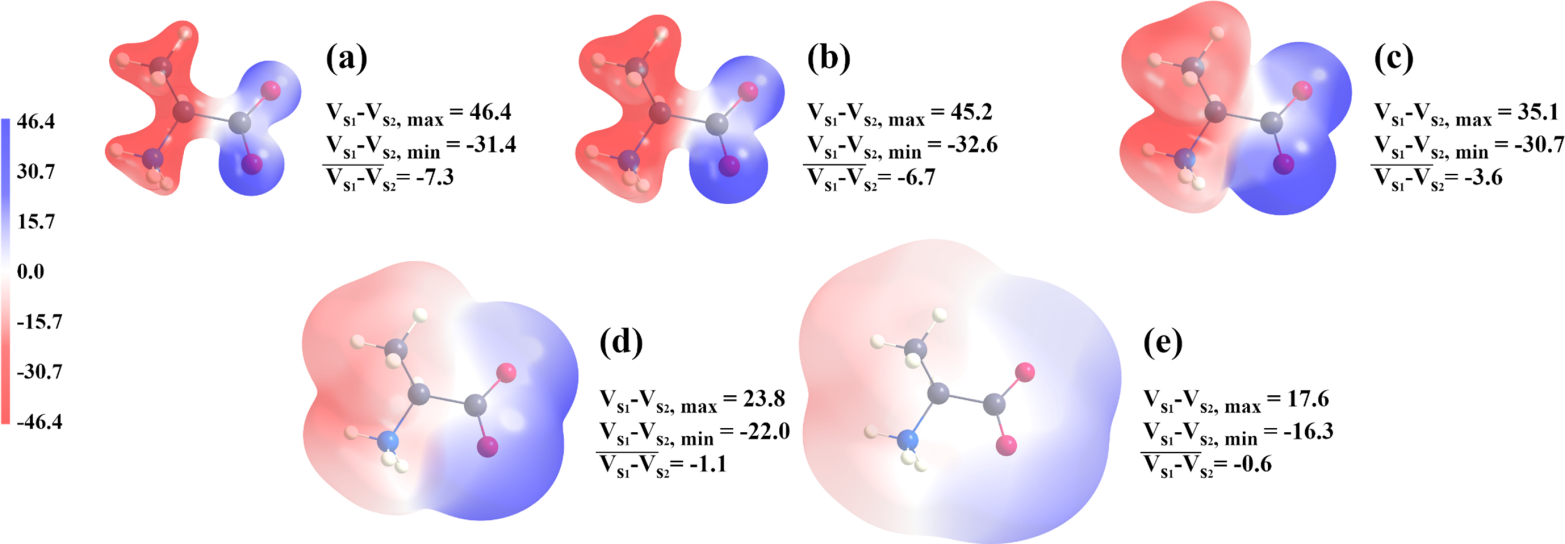
Visualization of the C_3_H_7_NO_2_ molecule iso-density surface with the difference between the reference electrostatic potential and the electrostatic potential of an MM based on the Def2TZVP basis set mapped onto it. Iso-density surfaces values are (*a*) 0.1 a.u., (*b*) 0.05 a.u., (*c*) 0.01 a.u., (*d*) 0.001 a.u. and (*e*) 0.0001 a.u.. The range of electrostatic potential values is from −46.4 to 46.4 kcal mol^−1^ e^−1^. *V*_s1_ − *V*_s2,max_, *V*_s1_ − *V*_s2,min_ and 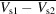 denote the maximum, minimum and average differences between electrostatic potentials on a given surface, respectively.

As was mentioned in Section 2.5, we examined the average electrostatic potential on five molecular surfaces, and before moving on to quantitative analysis we note that the closer the molecular surface is to the nucleus (and therefore the higher the energy of the surface), the greater the absolute values of electrostatic potential mapped onto this surface (Fig. 9 ▸). Also, regardless of the basis set the MM is based on and regardless of the molecule we consider, larger absolute values of electrostatic potential are associated with larger differences between the MM and the reference (an example is shown in Fig. 10 ▸).

**Figure 11:**
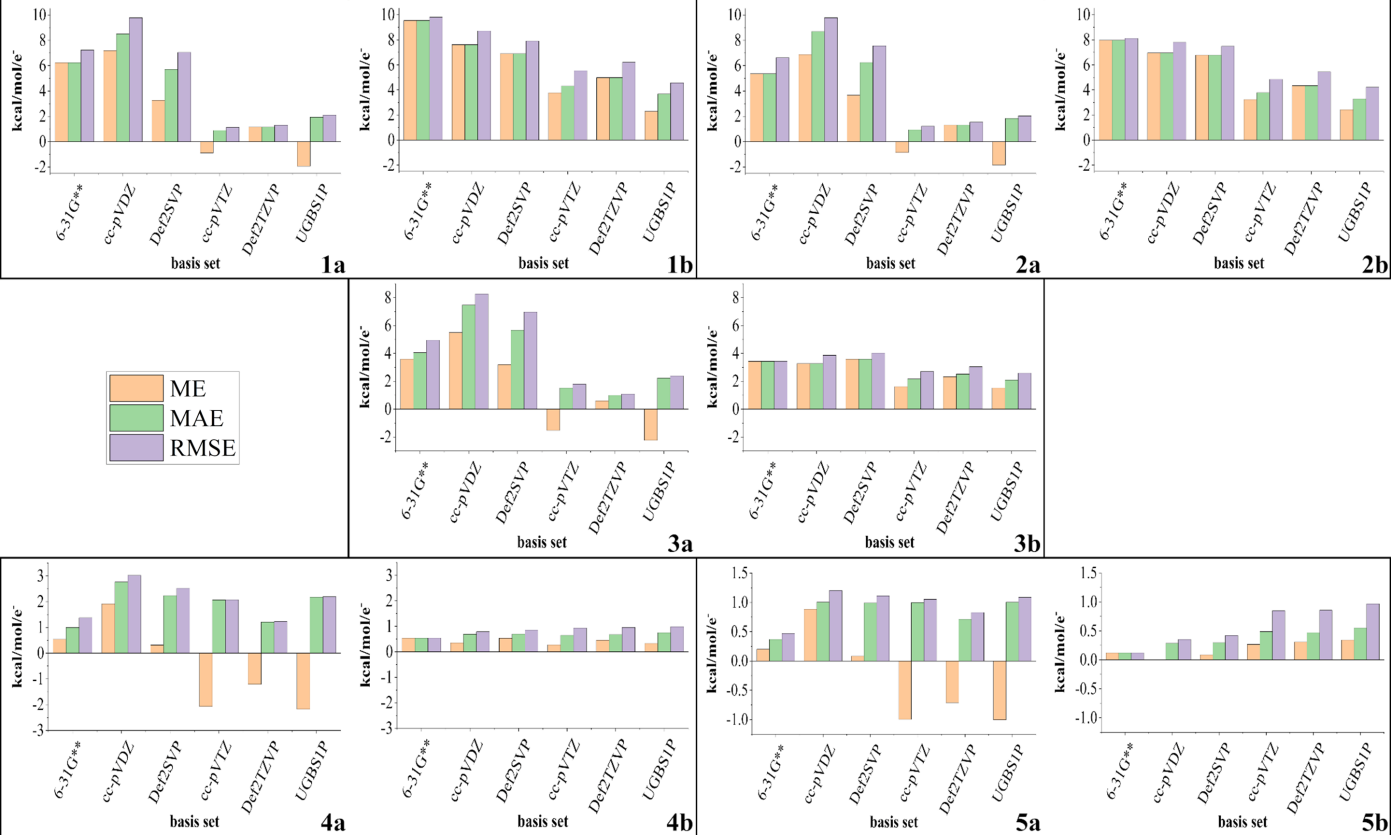
Average errors in MM electrostatic potential values averaged over a certain molecular surface. Plots labelled with the suffix (*a*) are for intermolecular complexes of magnesium and those with the suffix (*b*) are for the rest of the molecules. Iso-density surface values: (1) 0.1 a.u., (2) 0.05 a.u., (3) 0.01 a.u., (4) 0.001 a.u. and (5) 0.0001 a.u. ME denotes mean error, MAE denotes mean absolute error and RMSE denotes root-mean-square error (Section 2.5). Graphs for the same molecular surface are given on the same scale.

The average errors in the electrostatic potential values averaged over the molecular surface (Fig. 11 ▸) show that, regardless of the sample being considered (complexes of magnesium or molecules with covalent bonds only), the application of double-zeta basis sets results in bigger errors in the average electrostatic potential on high-energy molecular surfaces (0.1, 0.05 and 0.01 a.u.; Fig. 11 ▸, top and middle rows) than are obtained with bigger basis sets. For the intermolecular complexes of magnesium this gap in errors between double-zeta and bigger basis sets is larger. However, when we move to lower-energy molecular surfaces this difference gradually decreases. For low-energy molecular surfaces (0.001 and 0.0001 a.u.; Fig. 11 ▸, bottom row), in some cases application of double-zeta basis sets results in smaller errors in the average electrostatic potential.

**Figure 12:**
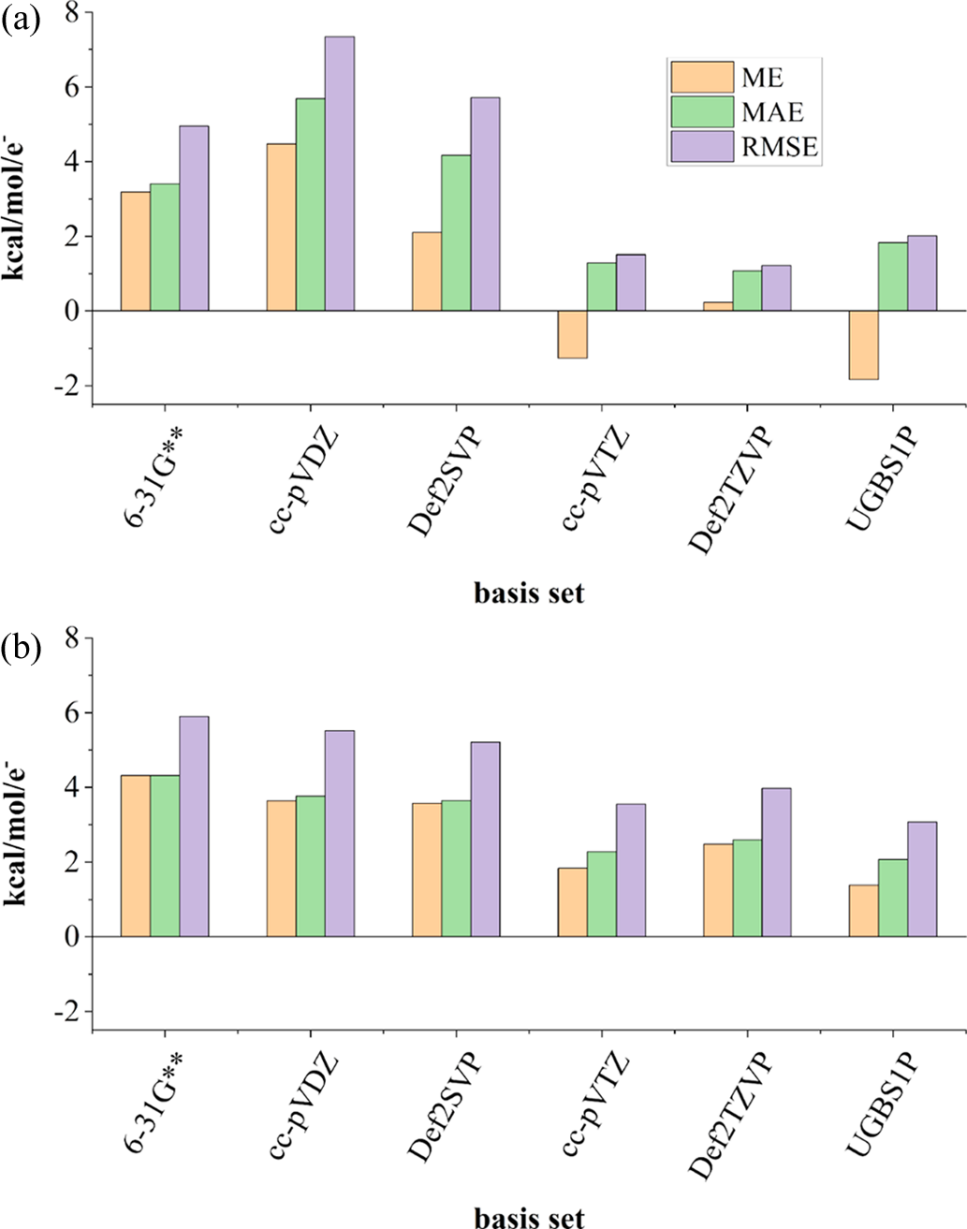
Average errors of MM electrostatic potential values averaged over all molecular surfaces under study, (*a*) for intermolecular complexes of magnesium and (*b*) for the rest of the molecules. ME denotes mean error, MAE denotes mean absolute error and RMSE denotes root-mean-square error (Section 2.5). Graphs are given on the same scale.

Although triple-zeta basis sets and UGBS1P do not outperform smaller basis sets in terms of electrostatic potential in all the sections of space around the molecule, they result in smaller errors in the average electrostatic potential in high-energy regions, which make the biggest contribution to the total electrostatic potential of the molecule. This is illustrated in Fig. 12 ▸. In both cases (complexes of magnesium or mol­ecules with covalent bonds only), when we consider all molecular surfaces under study together, bigger basis sets outperform double-zeta basis sets in terms of averaged electro­static potential error, *i.e.* average errors are dominated by surfaces close to the nuclei.

We also note that electrostatic potentials computed from MMs follow quite well the trends observed for electrostatic potentials computed directly from wavefunctions [Section S2 (Figs. S9 and S10) in the supporting information].

### 3.3. Aspherical features of MM and original wavefunction

**Figure 13:**
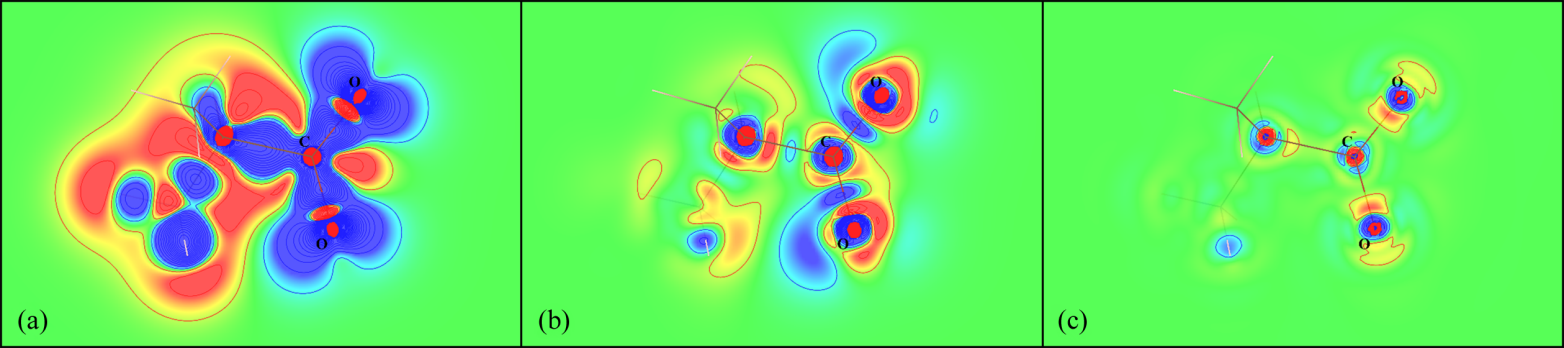
Differences between the electron densities of different models in the O—C—O plane of the C_3_H_7_NO_2_ molecule. (*a*) The electron density of the reference wavefunction minus the electron density of the IAM (deformation electron density, ρ_ref_ − ρ_IAM_). (*b*) The electron density of the reference wavefunction minus the electron density of an MM based on the Def2TZVP basis set (ρ_ref_ − ρ_MM_). (*c*) The electron density of the reference wavefunction minus the electron density of a wavefunction in the Def2TZVP basis set (ρ_ref_ − ρ_wf_). Contour levels are 0.05 e Å^−3^. Blue indicates positive regions and red indicates negative regions. Electron densities are visualized using the *VESTA* program (Momma & Izumi, 2011 ▸).

Although our work is focused on the electrostatic potential modelled using an MM, in order to illustrate a general idea of the differences between the models we have considered, we think it is necessary to refer to non-spherical features of electron density (Fig. 13 ▸). As can be seen in the example of the C_3_H_7_NO_2_ molecule, an MM [ρ_ref_ − ρ_MM_, Fig. 13 ▸(*b*)] cannot ‘recreate’ all the features of electron density of the original wavefunction [ρ_ref_ − ρ_wf_, Fig. 13 ▸(*c*)]. However, it is still much more advanced than the spherical approach [ρ_ref_ − ρ_IAM_, Fig. 13 ▸(*a*)].

**Figure 14:**
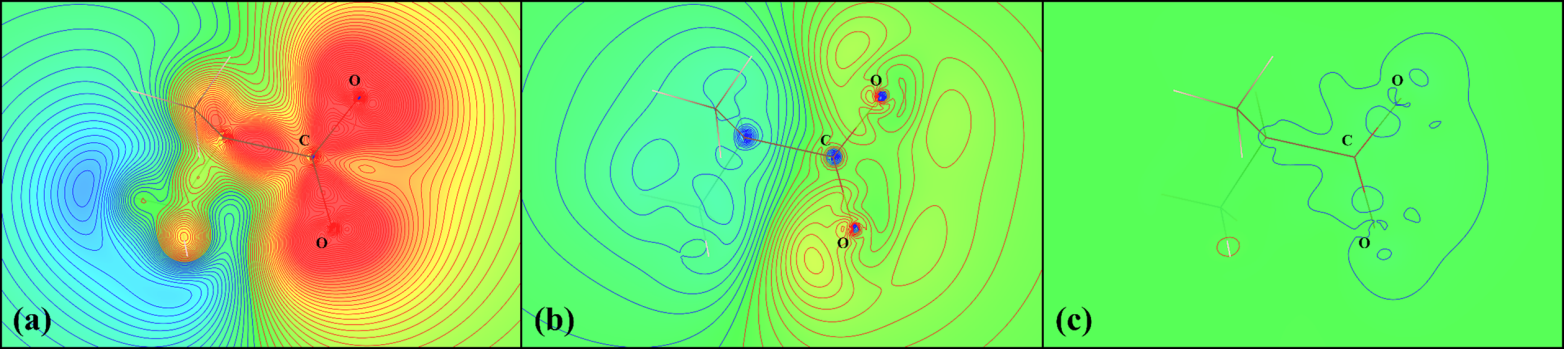
Differences between the electrostatic potential of different models in the O—C—O plane of the C_3_H_7_NO_2_ molecule. (*a*) The electrostatic potential of the reference wavefunction minus the electrostatic potential of the IAM (deformation electrostatic potential, *V*_ref_ − *V*_IAM_). (*b*) The electrostatic potential of the reference wavefunction minus the electrostatic potential of an MM based on the Def2TZVP basis set (*V*_ref_ − *V*_MM_). (*c*) The electrostatic potential of the reference wavefunction minus the electrostatic potential of a wavefunction in the Def2TZVP basis set (*V*_ref_ − *V*_wf_). Contour levels are 4 × 10^−3^ e Å^−1^. Blue indicates positive regions and red indicates negative regions. Electrostatic potentials are visualized using *VESTA* (Momma & Izumi, 2011 ▸).

The same trend holds for the electrostatic potential of the same molecule (Fig. 14 ▸), which is directly dependent on the electron density. Example of differences between MMs based on different basis sets are provided in Section S4 in the supporting information.

Under this topic, we mention a study which partially overlaps with ours. Koritsanszky *et al.* (2012 ▸) warn that an MM fitted to the wavefunction may be severely biased or even meaningless if the wavefunction is calculated using too extended a basis set. In some cases, the multipole parameters fitted to such a wavefunction may exhibit an electron density difference between the MM and the wavefunction comparable to the electron density difference between spherical atoms and the wavefunction, thus making such an interpretation of aspherical electron density meaningless. However, to calculate the theoretical electron density the authors used methods including electron correlation (BLYP, B3LYP or MP2) and basis sets of at least triple-zeta quality (cc-pVTZ, aug-cc-pVTZ or QZ4P). It is also important to note that Koritsanszky *et al.* (2012 ▸) used all-electron theoretical structure factors for MM fitting, while we use valence-only theoretical structure factors.

When fitting an MM to a wavefunction, we should keep in mind the imperfections of the MM. Nevertheless, our work shows that, in studies requiring fitting of an MM to theoretical structure factors, for the wavefunction calculation stage it is important to use basis sets of at least triple-zeta quality, since the MM’s charge density benefits from it.

## 4. Conclusions

In this work we have investigated the quality of charge density obtained by fitting of an MM to theoretical structure factors based on wavefunctions in different basis sets. The complex analysis of the correlation between electrostatic potentials, atomic electron populations and electrostatic potential values averaged over molecular surfaces shows that the size of the basis set used for the wavefunction calculation stage influences the charge density related properties, and switching from double-zeta to higher-zeta basis sets has a positive impact on an MM’s charge density. The degree of improvement depends on the structure under study and the most notable improvement is observed in the case of intermolecular complexes of magnesium. Since the results of multipolar refinement depend on the type of radial function used, it is important to mention that within our approach the usage of wavefunctions in basis sets of quality higher than double-zeta improves the results of switching from the CR to the SC radial function data bank.

With all that said, in order to model the charge density of a molecule properly, we recommend the use of basis sets of no lower than triple-zeta quality in studies involving the fitting of an MM to theoretical structure factors based on DFT calculations. This is particuarly true if the goal is to achieve low errors in all regions of the spatial distribution of the charge density, which is important for the interpretation of diffraction data.

This research enables the creation of a new version of the MATTS data bank, which will be expanded to include atom types for elements heavier than Kr and selected metal complexes important for biological systems.

## Supplementary Material

Additional discussion, figures and table. DOI: 10.1107/S1600576724009841/ui5014sup1.pdf

## Figures and Tables

**Figure 8:**
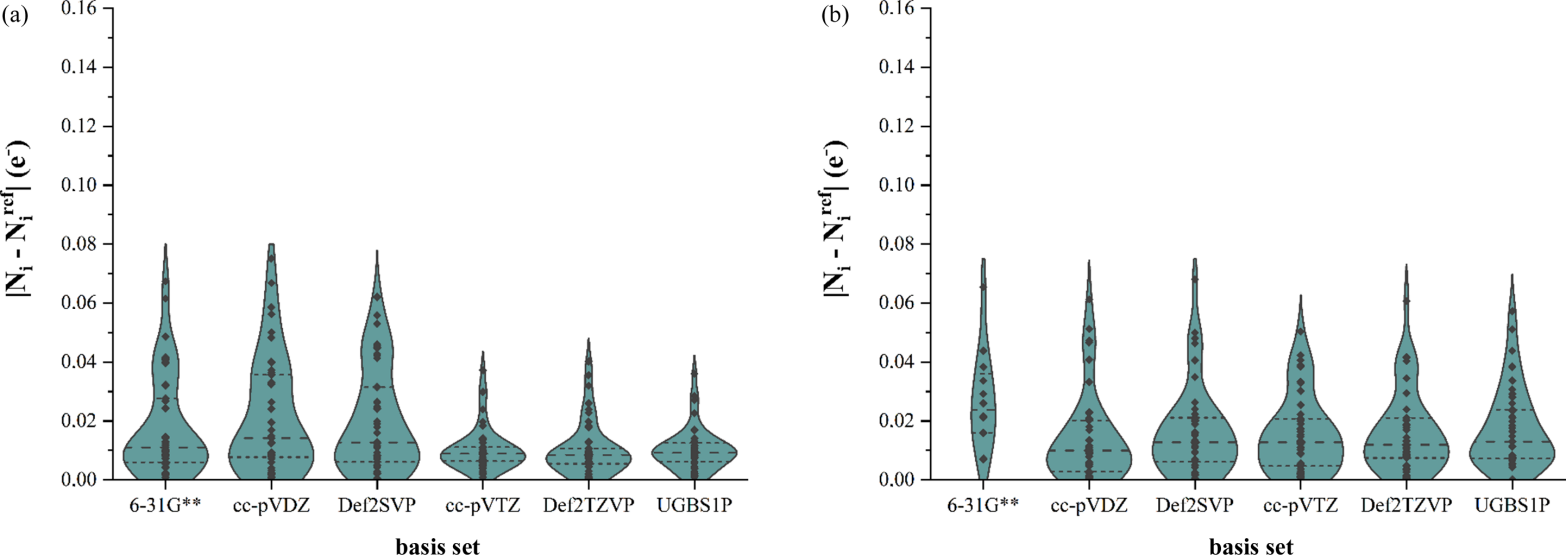
Errors in hydrogen atom electron populations of MMs based on different basis sets. (*a*) Intermolecular complexes of magnesium and (*b*) the rest of the molecules.

**Table 1:** Chosen features of the basis sets under study

	6-31G**	cc-pVDZ-PP/cc-pVDZ	Def2SVP	cc-pVTZ-PP/cc-pVTZ	Def2TZVP	UGBS1P
No. of basis functions for the C_6_H_5_Cl molecule	134	127	127	284	253	1623
Elements available	H–Kr	H–Rn	H–Rn	H–Rn	H–Rn	H–Ra (except Pa, U and Np)
Effective core potential applied to	–	Cu and heavier	Rb and heavier	Cu and heavier	Rb and heavier	–
Effective core potential size	–	All shells except valence shell and two preceding shells	All shells except valence shell and preceding shell	All shells except valence shell and two preceding shells	All shells except valence shell and preceding shell	–
Computational cost	Low	Low	Low	Medium	Medium	Extremely high
