# Supplementary material for: Towards expansion of the MATTS data bank with heavier elements: the influence of the wavefunction basis set on the multipole model derived from the wavefunction
Source: J Appl Crystallogr. 2024 Nov 17;57(Pt 6):1884–95. doi: 10.1107/S1600576724009841 (PMC12418678; doi:10.1107/S1600576724009841)
Supplement: Supplementary file 1 [file j-57-01884-sup1.pdf]

JOURNAL OF APPLIED CRYSTALLOGRAPHY

Volume 57 (2024)

Supporting information for article:

Towards expansion of the MATTS data bank with heavier elements: the influence of the wavefunction basis set on the multipole model derived from the wavefunction

Vladislav Ignat'ev and Paulina Maria Dominiak

S1. Influence of hydrogen atoms multipole expansion on multipole refinement

In section 2.1 we noted that during the multipole refinement stage we truncated the hydrogen atoms multipole expansion up to quadrupoles. We apply such a restriction in routine generation of MATTS databank (Rybicka et al., 2022; Jha et al., 2022) data. There are two reasons for this. First, high computational cost of refinement with high-order multipoles on hydrogen atoms. Second, MATTS-based refinement of X-ray experimental data do not benefit from such a detailed description of hydrogen atoms. However, we want to show that application of the hydrogen atoms multipole expansion up to hexadecapoles in the framework of this study does not change the trends highlighted in results and discussion section, and therefore does not change the conclusion (Figures S1, S2, S3 and S4).

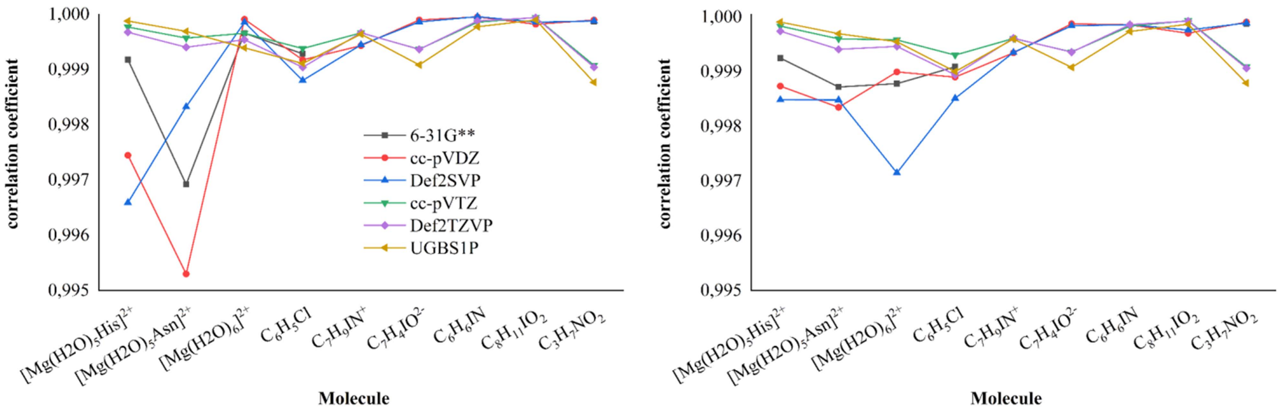

(a) (b)

Figure S1 Pearson correlation coefficients between electrostatic potential of multipole model based on wave function in particular basis set and electrostatic potential of wave function in UGBS1P basis set (**a** - hydrogen atoms multipole expansion up to quadrupoles, **b** - hydrogen atoms multipole expansion up to hexadecapoles).

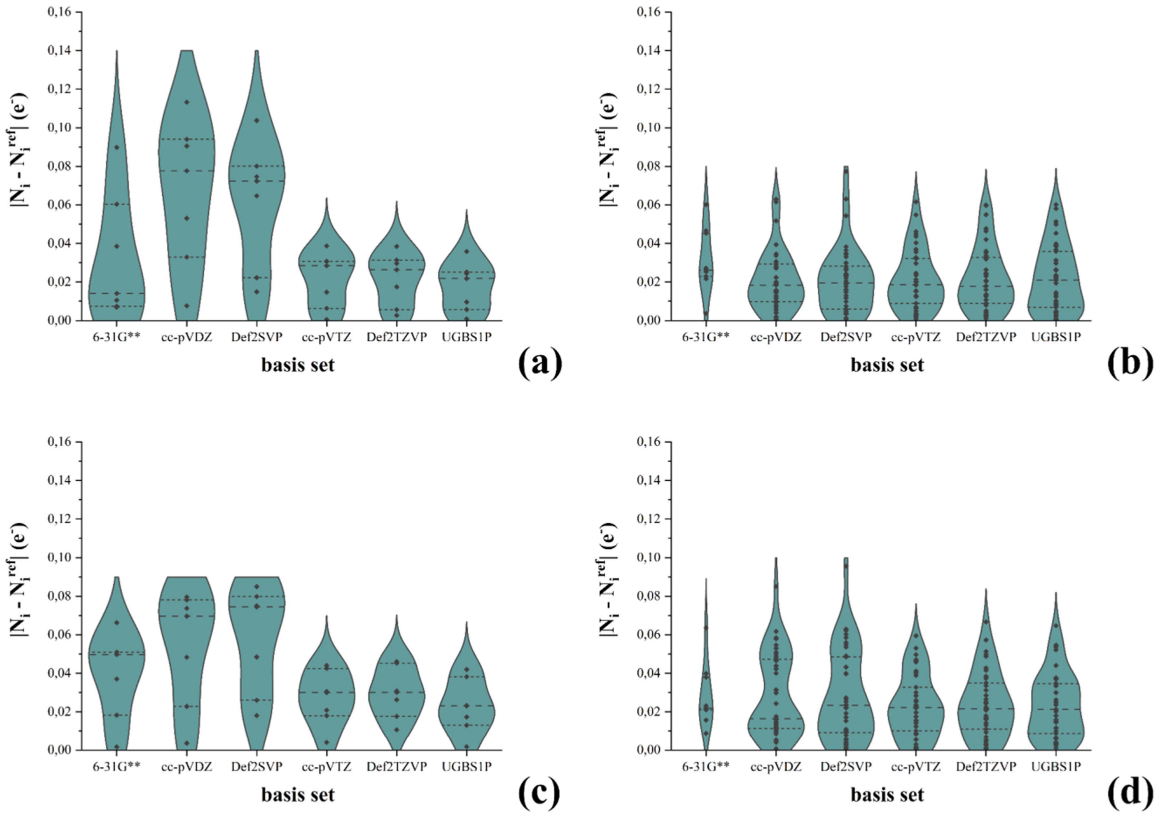

Figure S2 Errors of carbon atoms electron populations of multipole models based on different basis sets (**a** - intermolecular complexes of magnesium, hydrogen atoms multipole expansion up to quadrupoles; **b** – rest of the molecules, hydrogen atoms multipole expansion up to quadrupoles; **c** - intermolecular complexes of magnesium, hydrogen atoms multipole expansion up to hexadecapoles; **d** rest of the molecules, hydrogen atoms multipole expansion up to hexadecapoles).

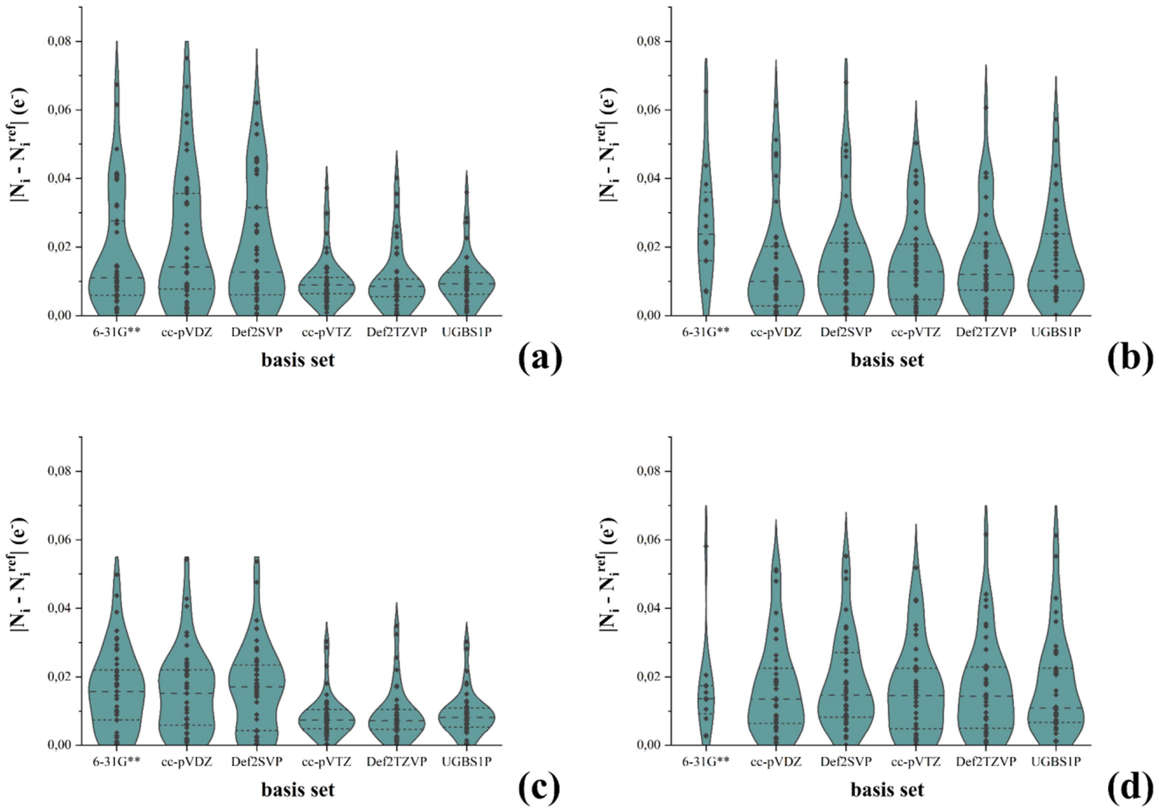

Figure S3 Errors of hydrogen atoms electron populations of multipole models based on different basis sets (**a** - intermolecular complexes of magnesium, hydrogen atoms multipole expansion up to quadrupoles; **b** – rest of the molecules, hydrogen atoms multipole expansion up to quadrupoles; **c** - intermolecular complexes of magnesium, hydrogen atoms multipole expansion up to hexadecapoles; **d** rest of the molecules, hydrogen atoms multipole expansion up to hexadecapoles).

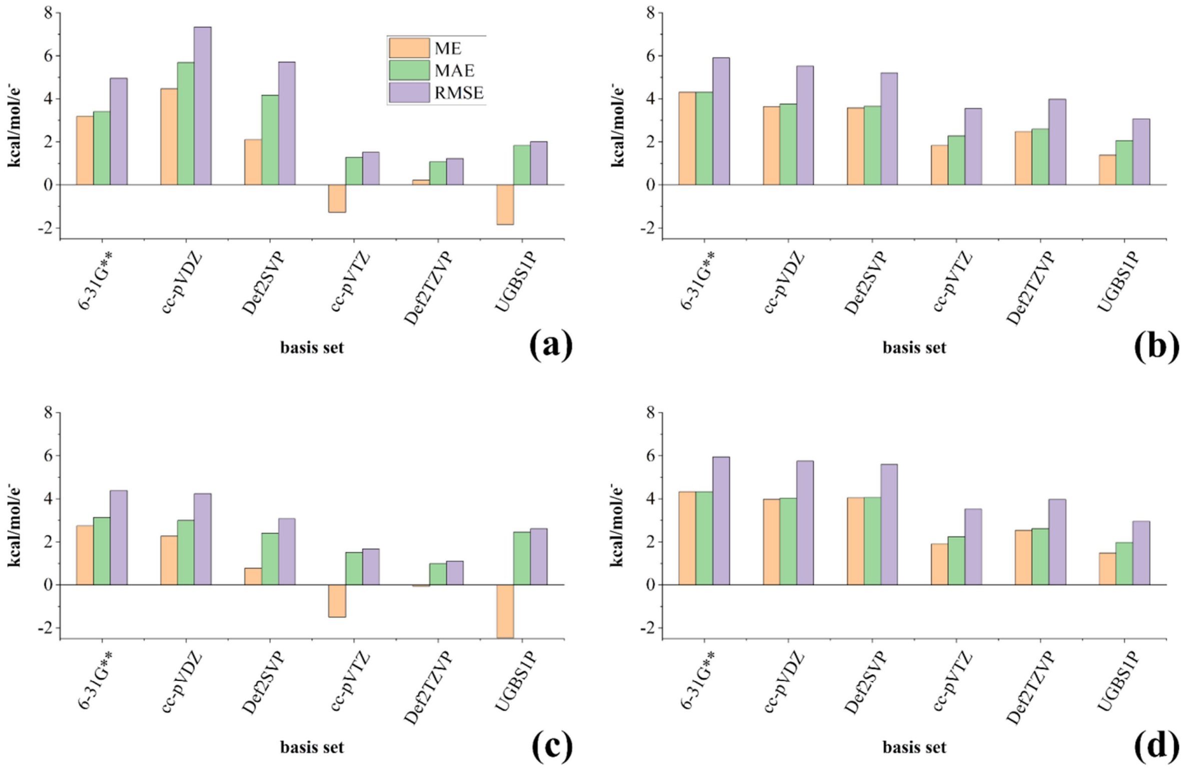

Figure S4 Average errors of multipole models electrostatic potential values averaged over all molecular surfaces under study (**a** - intermolecular complexes of magnesium, hydrogen atoms multipole expansion up to quadrupoles; **b** – rest of the molecules; hydrogen atoms multipole expansion up to quadrupoles; **c** - intermolecular complexes of magnesium, hydrogen atoms multipole expansion up to hexadecapoles; **d** rest of the molecules, hydrogen atoms multipole expansion up to hexadecapoles; ME – Mean Error, MAE – Mean Absolute Error, RMSE – Root Mean Square Error; see section 2.5).

S2. Difference between wave functions in different basis sets

Despite the fact that it goes beyond our study, we think that some readers might be interested to look at the difference between wave functions, to which we fitted multipole model. To link this chapter with the main subject of the paper, we considered wave functions from the charge density properties point of view. If we will take a look at the correlation coefficients between electrostatic potentials (Figure S5, Table S1 and S2), we will see that in most of the cases correlation between wave functions and reference (wave function in UGBS1P basis set) is much higher than correlation between resulting multipole model and reference. Exceptions are molecules containing iodine atoms ($C_7H_9IN^+$, $C_7H_4IO_2^-$, C_6H_6IN and $C_8H_{11}IO_2$). We assume that the reason is the effective core potential presented in all wave functions of these molecules, except the reference wave function (introduction, Table 1). However, the aforementioned “flaw” of electrostatic potential of wave functions using effective core

potential does not influence the multipole models built within our approach, since we exclude molecular orbitals of core electrons at the theoretical structure factors calculation stage (see section 2.1).

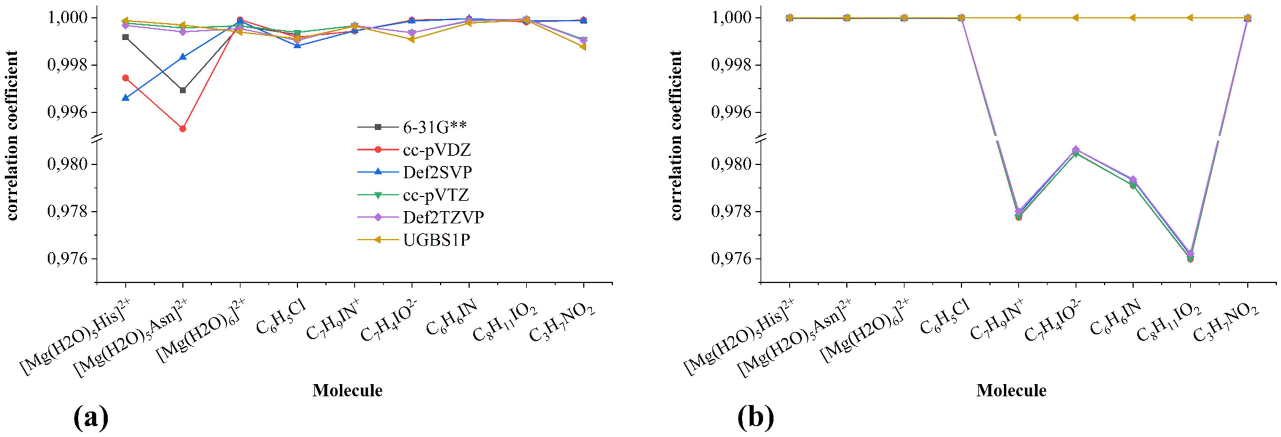

Figure S5 Pearson correlation coefficients between electrostatic potential of multipole model based on wave function in particular basis set and electrostatic potential of wave function in UGBS1P basis set (**a**); Pearson correlation coefficients between electrostatic potential of wave function in particular basis set and electrostatic potential of wave function in UGBS1P basis set (**b**) ; graphs are given on the same scale.

Table S1 Pearson correlation coefficients between electrostatic potential of multipole model based on wave function in particular basis set and electrostatic potential of wave function in UGBS1P basis set.

	6-31G**	cc-pVDZ	Def2SVP	cc-pVTZ	Def2TZVP	UGBS1P
[Mg(H2O)5His]2+	0.99918	0.99745	0.99659	0.99977	0.99968	0.99988
[Mg(H2O)5Asn]2+	0.99692	0.99530	0.99833	0.99957	0.99940	0.99969
[Mg(H2O)6]2+	0.99966	0.99991	0.99986	0.99966	0.99954	0.99939
C6H5Cl	0.99928	0.99918	0.99880	0.99938	0.99904	0.99911
C7H9IN+		0.99943	0.99945	0.99966	0.99967	0.99964
C7H4IO2-		0.99990	0.99986	0.99937	0.99936	0.99909
C6H6IN		0.99995	0.99996	0.99986	0.99988	0.99978
C8H11IO2		0.99982	0.99986	0.99994	0.99994	0.99990
C3H7NO2	0.99987	0.99990	0.99988	0.99908	0.99904	0.99877

Table S2 Pearson correlation coefficients between electrostatic potential of wave function in particular basis set and electrostatic potential of wave function in UGBS1P basis set.

	6-31G**	cc-pVDZ	Def2SVP	cc-pVTZ	Def2TZVP	UGBS1P
[Mg(H2O)5His]2+	0.99998	0.99999	0.99999	1.00000	1.00000	1.00000
[Mg(H2O)5Asn]2+	0.99998	0.99999	0.99999	1.00000	1.00000	1.00000
[Mg(H2O)6]2+	0.99997	0.99999	0.99998	1.00000	1.00000	1.00000
C6H5Cl	0.99998	0.99999	0.99999	1.00000	1.00000	1.00000
C7H9IN+		0.97775	0.97790	0.97780	0.97801	1.00000
C7H4IO2-		0.98048	0.98063	0.98046	0.98063	1.00000
C6H6IN		0.97910	0.97934	0.97911	0.97937	1.00000
C8H11IO2		0.97599	0.97616	0.97601	0.97622	1.00000
C3H7NO2	0.99994	0.99994	0.99995	0.99999	1.00000	1.00000

Another interesting observation associated with electrostatic potential correlation coefficients of wave functions which do not use effective core potential (wave functions of molecules with no heavy elements: $[Mg(H2O)_5His]^{2+}$, $[Mg(H2O)_5Asn]^{2+}$, $[Mg\ (H2O)_6]^{2+}$, C_6H_6Cl and $C_3H_7NO_2$) is that they showed the gap between double-zeta and bigger basis sets similar to what we discussed in results and discussion section (but on a much smaller scale, Figure S6).

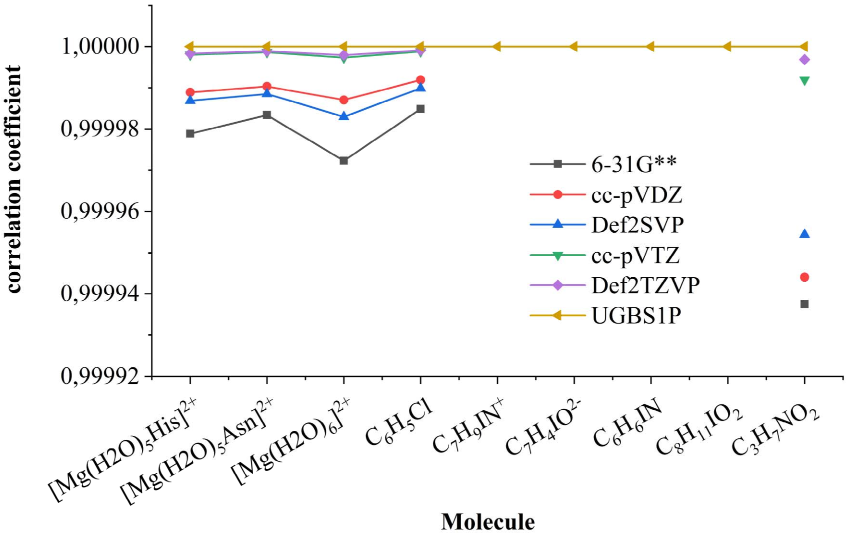

Figure S6 Pearson correlation coefficients between electrostatic potential of wave functions with no effective core potential and electrostatic potential of wave function in UGBS1P basis set.

When we examine atomic electron populations of wave functions themselves, we can see that while carbon atoms do not follow the trends of multipole model's atomic electron populations (Fig. S7), the

hydrogen atoms show a clear dependence on the wave function's basis set size (Fig. S8). Yet, it is worth noting that magnitude of errors is much smaller than in case of multipole models.

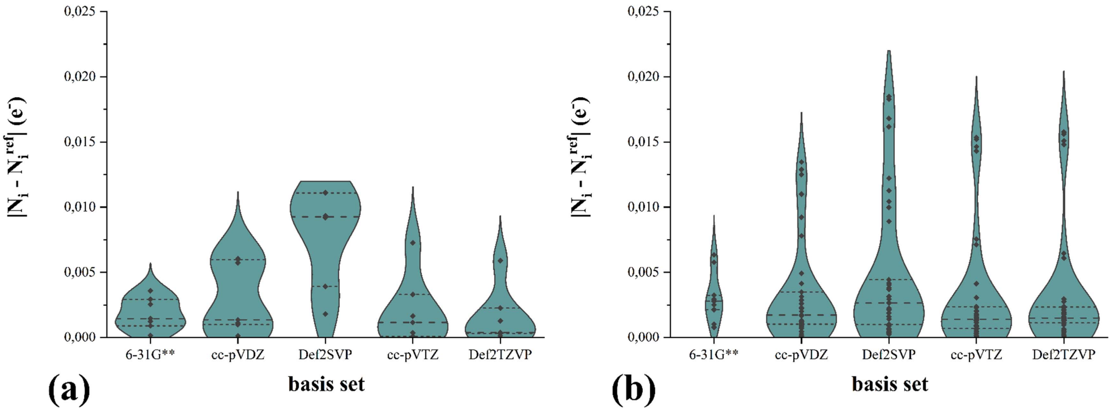

Figure S7 Errors of carbon atoms electron populations of wave functions based on specific basis sets (**a** - intermolecular complexes of magnesium; **b** – rest of the molecules).

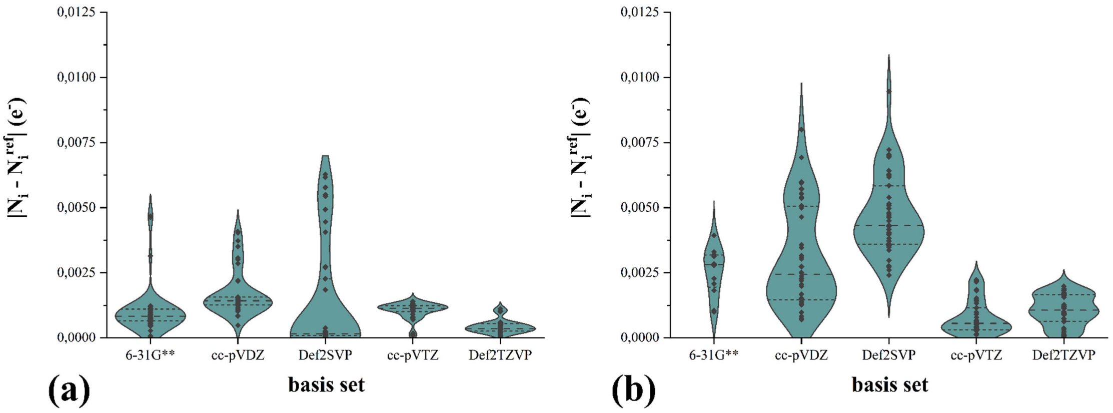

Figure S8 Errors of hydrogen atoms electron populations of wave functions based on specific basis sets (**a** - intermolecular complexes of magnesium; **b** – rest of the molecules).

The errors of electrostatic potential averaged over certain molecular surfaces (Figure S9) show that, as in the case of multipole models, wave functions in double-zeta basis sets offer larger average electrostatic potential error on high-energy surfaces and these surfaces make the biggest contribution to the total electrostatic potential (Figure S10).

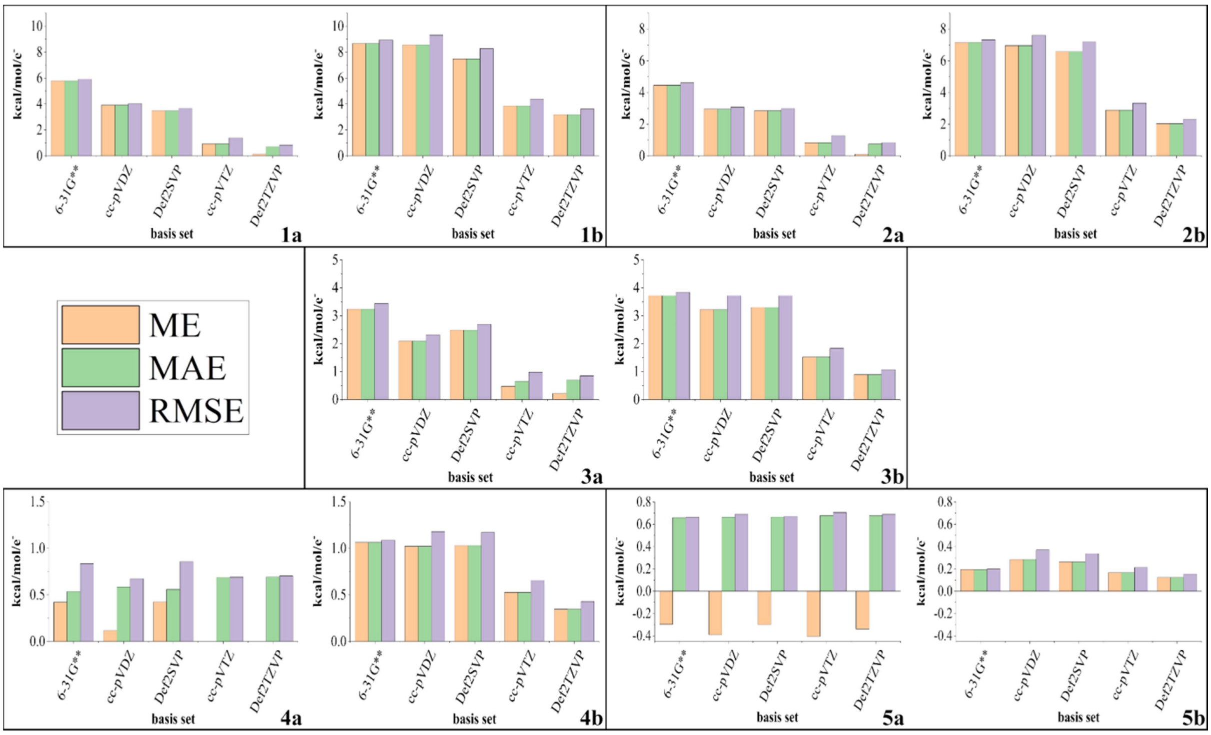

Figure S9 Average errors of wave functions electrostatic potential values averaged over certain molecular surface (**a** - intermolecular complexes of magnesium; **b** – rest of the molecules; iso-density surfaces values: **1** – 0.1 a.u., **2** – 0.05 a.u., **c** – 0.01 a.u., **3** – 0.001 a.u., **4** – 0.0001 a.u., **5** – 0.0001 a.u.; ME – Mean Error, MAE – Mean Absolute Error, RMSE – Root Mean Square Error; see section 2.5; graphs for the same molecular surface are given on the same scale).

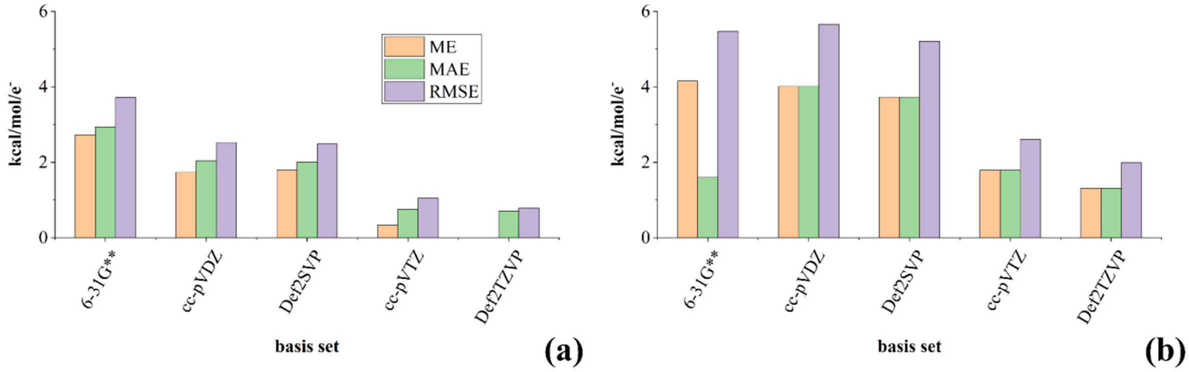

Figure S10 Average errors of wave functions electrostatic potential values averaged over all molecular surfaces under study (**a** - intermolecular complexes of magnesium, **b** – rest of the molecules; ME – Mean Error, MAE – Mean Absolute Error, RMSE – Root Mean Square Error; see section 2.5).

S3. Atomic electron populations of elements rarely found in samples

When comparing atomic electron populations of multipole model based on wavefunction in specific basis set and reference atomic electron populations we focused on electron populations of hydrogen and carbon atoms (see Fig. 7 and 8 in results and discussion section). Other elements are found much less frequently in the molecules under study; therefore, they have lower statistical power. However, we decided to show the graphs of electron populations errors related to nitrogen, oxygen, magnesium, chlorine and iodine here:

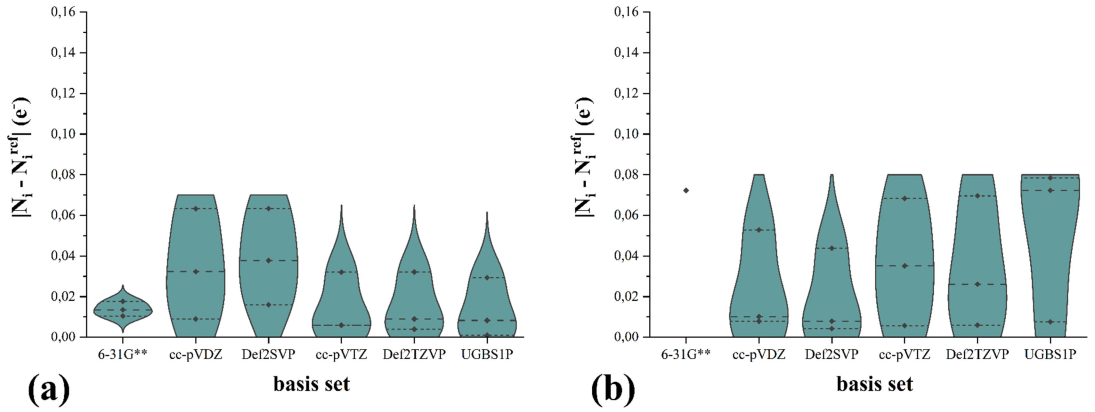

Figure S11 Errors of nitrogen atoms electron populations of multipole models based on different basis sets (**a** - intermolecular complexes of magnesium; **b** – rest of the molecules)

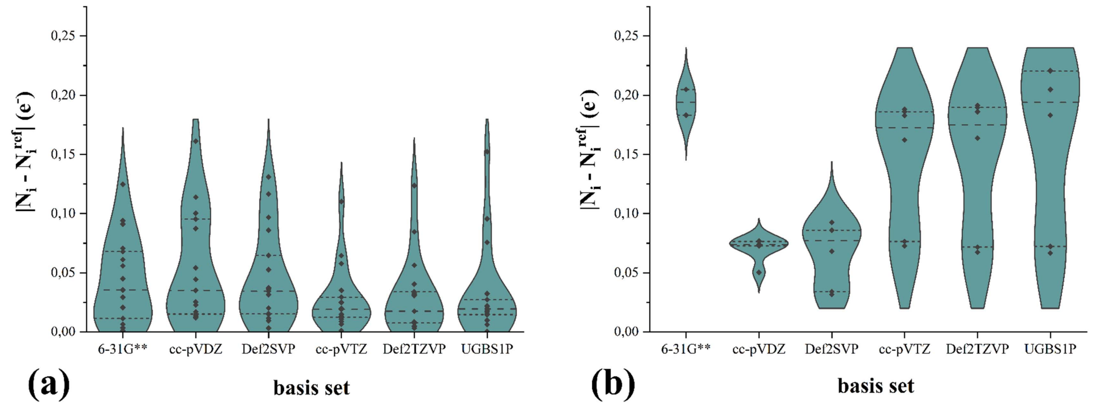

Figure S12 Errors of oxygen atoms electron populations of multipole models based on different basis sets (**a** - intermolecular complexes of magnesium; **b** – rest of the molecules)

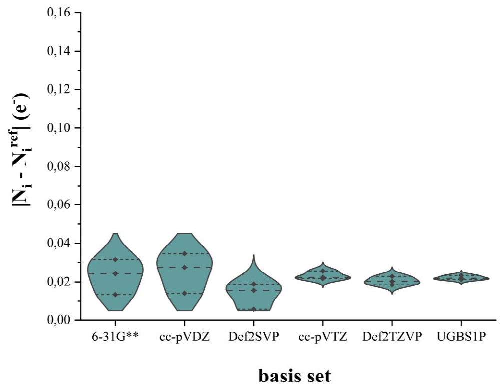

Figure S13 Errors of magnesium atoms electron populations of multipole models based on different basis sets

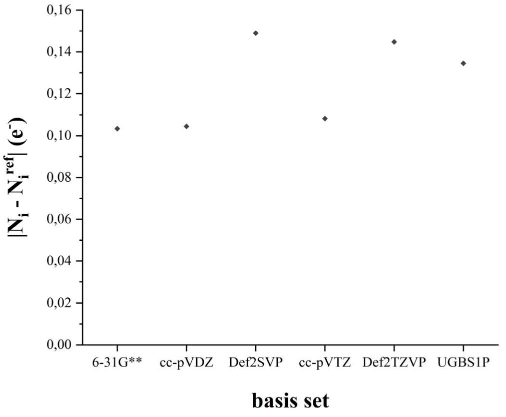

Figure S14 Errors of chlorine atoms electron populations of multipole models based on different basis sets

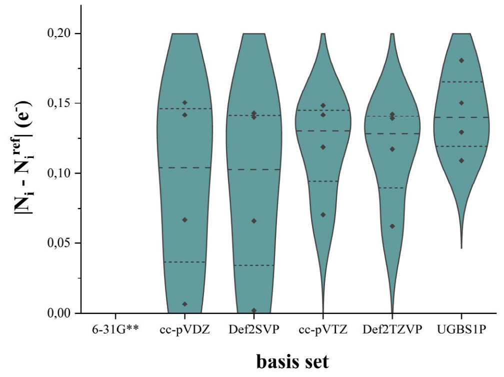

Figure S15 Errors of iodine atoms electron populations of multipole models based on different basis sets

S4. Deformation density of multipole models based on double- and triple-zeta basis sets

In the main body of the work, we demonstrated the difference between multipole models based on wavefunctions in different basis sets using averaged charge density-related properties. However, maybe some of the readers would be interested in not quantitative, but qualitative (more "illustrative") example. Here we provide the pictures of deformation electron density and deformation electrostatic potential (Figure S16). As we can see, the difference is quite small, but still visible at this contour level.

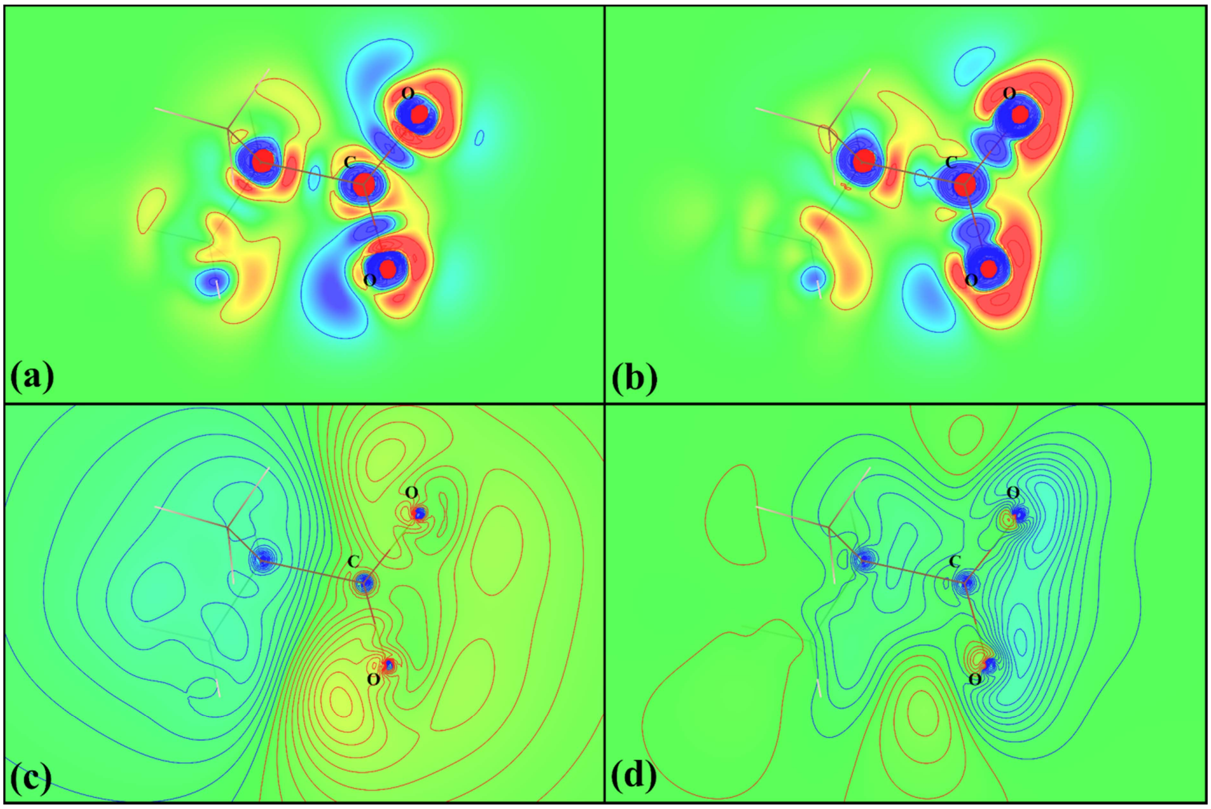

Figure S16 Difference between electron density (**a** – electron density of reference wave function minus electron density of multipole model based on Def2TZVP basis set, **b** - electron density of reference wave function minus electron density of multipole model based on 6-31G** basis set) and electrostatic potential (**c** - electrostatic potential of reference wave function minus electrostatic potential of multipole model based on Def2TZVP basis set, **d** - electrostatic potential of reference wave function minus electrostatic potential of multipole model based on 6-31G** basis set) of different models in O-C-O plane of $C_3H_7NO_2$ molecule (contour levels are 0.05 e/Å^3 for electron densities and $4 \cdot 10^{-3}$ e/Å for electrostatic potentials; blue – positive regions, red – negative regions; visualized using VESTA program (Momma & Izumi, 2011))).
